# Different types of voltametric techniques for selective and sensitive detection of heavy metal ions in wastewater system: a review

**DOI:** 10.1039/d6ra02188c

**Published:** 2026-07-02

**Authors:** Md. Shadat Hossain, Md. Habibur Rahman, Md. Tariqur Rahaman Bhuiyan Mamun, Md. Kawcher Alam, Md. Sahadat Hossain

**Affiliations:** a Department of Applied Chemistry and Chemical Engineering, Noakhali Science and Technology University Noakhali Bangladesh kawcherarif00@gmail.com; b Institute of Glass & Ceramic Research and Testing, Bangladesh Council of Scientific and Industrial Research (BCSIR) Dhaka 1205 Bangladesh saz8455@gmail.com

## Abstract

Heavy metal contamination in wastewater poses serious environmental and health risks, for which highly selective and sensitive detection methods are required. Voltametric techniques, such as DPASV, SWASV, ASV, CV, and DPV, offer simultaneous detection of multiple elements with quick analysis, accuracy and exceptional sensitivity, good selectivity, and least sample requirements. These techniques determine one or more analytes are measured by observing the current with potential. Among them, those that introduce a preconcentration step have been proven to be powerful electroanalytical techniques for detecting trace metals such as SWASV and DPASV. The outcome of experimental parameters, supporting electrolytes, and working electrodes and their impact on analytical performance are key focuses of this review. Numerous studies have explored voltammetric techniques for detecting heavy metals; however, a thorough comparison of their experimental parameters and analytical performance is uncommon. Operational conditions such as preconcentration voltage, deposition time, scan rate, pH, pulse amplitude, and supporting electrolyte play a crucial role in the magnification of the sensitivity and selectivity of the method. The optimal operational conditions were found to significantly improve the efficiency and analytical performance of the methods. Among the voltammetric techniques, SWASV offers enhanced sensitivity and reduced background noise, making it suitable for trace analysis. Results indicate that SWASV exhibits the lowest LOD in the ng L^−1^ range (*e.g.*, 0.001 µg L^−1^ for Pb^2+^). DPASV and ASV are also commonly used for heavy metal detection. SWASV and DPASV are the most widely used methods for HMIs determination, while CV is more suitable for qualitative electrochemical characterization rather than trace-level quantification. This review serves as a valuable resource for researchers optimising voltammetric parameters for real-world wastewater applications.

## Abstract 2

Heavy metal contamination in wastewater poses serious environmental and health risks, for which highly selective and sensitive detection methods such as various voltametric techniques (DPASV, SWASV, ASV, CV and DPV) are required.

## Graphical abstract

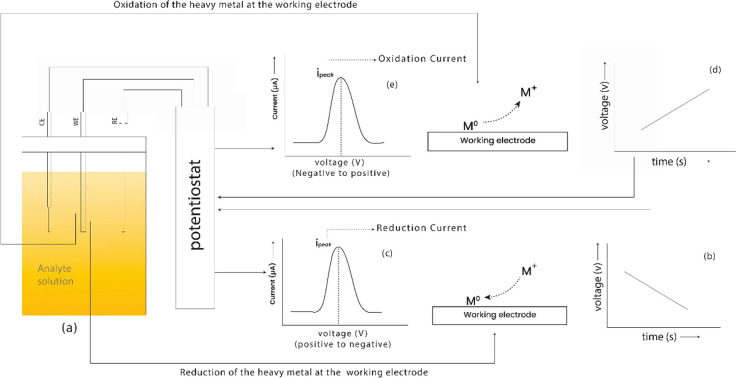

## Introduction

As urbanization rates in emerging nations rise and inhabitants pursue improved living conditions, more quantities of freshwater are allocated to home, banking, and industrial sectors, resulting in higher volumes of wastewater. Wastewater is often released into natural water bodies with minimal or no treatment, leading to significant pollution.^1–3^ And the quantity is expected to rise substantially due to population expansion and fast urbanization.^4,5^ This will lead to a drastic and continuous shortage of freshwater resources,^6^ which makes this matter one of the most significant environmental and public health issues of today.^7^ Wastewater is usually contaminated with trace elements, such as Cu, Cr, Zn, Pb, B, Co, As, Mo, and Mn; many of them are not essential and, with time, are harmful to plants, humans, and animals,^8^ soil, air, and water.^9^ Heavy metals like Cr, Cd, Hg, Pb, Ni, and Tl may be dangerous when in combination or as individual elements form. They dissolve easily in aquatic environments, and therefore, living organisms may easily absorb them.^10^ They can affect life systems directly or indirectly just by existing in aquatic environments. Because they are absorbed by plants and eventually make their way to animals and humans, they are particularly damaging to both plants and animals in the soil environment.^11^ Heavy metals are non-biodegradable constituents with relative atomic masses ranging from 63.5 to 200.6, atomic densities greater than 5 g cm^−3^, and the ability to directly impact living organisms and human health. They are quite persistent as well as stable in the water system.^12–15^ Elevated levels of these metals may enter the aquatic environment due to seeping from rocks, accumulation in the air, water flow, runoff from riverbanks, and the release of effluent from cities and factories.^16^ The heavy metals most frequently found in wastewater are toxic, and excessive discharge of these metals into water sources leads to significant health and environmental issues,^14^ owing to extreme toxicity, they are therefore regarded as one of the major environmental pollutants.^17^

Heavy metals are severely polluting environments worldwide, impacting the earth and marine environments with serious consequences for human health. Once in the biosphere, these metals persist^9^ and accumulate in marine species, impacting fish and invertebrates.^18^ Studies have found heavy metals in the tissues of fish in contaminated waters.^10^ Contamination from wastewater can degrade soil quality and microbial communities,^19^ while excess HMI's like Cd, Zn, Cu, Pb, and Ni can lower plant yields and affect food quality.^20^ These can enter the dietary chain through bioaccumulation, leading to health issues in humans,^21^ including cancer,^19^ organ damage, and neurological impairments.^15,21,22^ Long-term consumption of heavy metals, for example, As and Hg, results in significant health risks, as seen in cases like Minamata and itai–itai diseases.^22,23^ Thus, it is evident that these metals pose serious threats to the vitality of biological systems owing to their hazardous and perpetual characteristics.^24^ A high intake leads to severe outcomes, including carcinogenic effects,^25^ as well as various chronic and acute medical conditions such as hypertension,^26^ atherosclerotic disease, cardiovascular disease,^27^ renal failure, infertility, bone deformities,^28^ neurological diseases,^29^ prostate dysfunction, osteomalacia, and osteoporosis.^30^ Because of their excessive toxicity, heavy metals are harmful environmental pollutants. Industries such as metal processing, producing batteries, electrical wiring production, electronic processor fabrication, and extraction of minerals generate wastewater containing copper and cadmium.^17^ These pose substantial risks to the survival of all biological systems, given their perpetual hazardous and detrimental effects, as well as biological accumulation.^24^

Consequently, effluent containing metals must undergo remediation prior to environmental discharge.^31^ Before we remove these toxic HMIs from water samples, we must find a method to detect their presence. Therefore, a heavy metal detection method that is cost-effective, time-efficient, and environmentally benign needs to be developed. The conventional techniques for detecting heavy metals up to this point include inductively coupled plasma-optical emission spectrometry (ICP-OES),^32^ Atomic absorption spectroscopy (AAS).^33,34^ ICP-MS spectrometry (ICP-MS),^35^ X-ray fluorescence spectrometer (XRF)^36^ HPLC, coupled with electrochemical- or UV-Vis-detectors, flame atomic absorption spectrometry. These procedures, however, are costly and highly sensitive, demanding labor-intensive pre-treatment methods, and Most of these techniques require costly, advanced, and bulky equipment operated by trained personnel, which restricts *in situ* measurements.^37–40^ Whereas electrochemical approaches like voltammetric techniques are more economically efficient, time economical, easy to use, reliable, and suitable for practical applications. Comparing these electrochemical techniques to other spectroscopic techniques, they are quick, provide straightforward operations, and have a short analytical time. Additionally, they need less effort from the researcher.^38,39^

Voltammetry stands out as the sole electrochemical method for on-site monitoring and identification of HMI's, offering remarkable sensitivity.^41^ This approach has garnered considerable interest owing to its unique benefits in the analysis of these ions in complicated matrices. It enables the simultaneous detection of many elements with fast analysis, outstanding sensing and precision, enhanced selectivity, minimal specimen needs, and an extensive detection range. This approach facilitates exceptionally accurate *in situ* evaluation of HMIs. Additionally, its mobility and ease of use make it highly important for environmental monitoring and research.^42^ So many electrochemical techniques have been put in action to identify chemical biomolecules and pollutants.^43^ Normally, voltammetric methods, for instance, differential pulse voltammetry (DPV), cyclic voltammetry (CV), square wave anodic stripping voltammetry (SWASV), anodic stripping voltammetry (ASV), differential pulse anodic stripping voltammetry (DPASV), *etc.*, potentiometric techniques are employed in the sensing/detecting of analytes. Electrochemical methods incorporating a preconcentration phase, such as stripping voltammetry, have demonstrated efficacy as robust electroanalytical procedures for the identification of trace metals.^44^ Among these methods, the SWASV method has been tested and is recommended for both simultaneous and individual analysis of HMIs by various researchers.^45^

In this review, we focused on the five key voltammetric methods-square wave anodic stripping voltammetry, differential pulse voltammetry, differential pulse anodic stripping voltammetry, cyclic voltammetry, and anodic stripping voltammetry for heavy metal detection in wastewater by focusing on their experimental conditions and corresponding analytical performance. Although the use of voltammetric techniques for heavy metal detection has been documented in many studies and review papers, the majority of earlier research mostly concentrated on individual voltammetric techniques, particular heavy metals, or specific electrode modifications. There isn't yet a thorough comparison of the primary voltammetric methods for wastewater applications that focuses on optimum experimental settings, analytical performance, supporting electrolytes, working electrode materials, and detection capabilities. In contrast to earlier research, this effort focuses on: (i) evaluating voltammetric methods in comparison; (ii) optimizing operating parameters; (iii) applications focused on wastewater; and (iv) new developments in electrode materials and ultra-trace heavy metal detection. Here, we have compiled recent experimental data, mostly from 2001 to 2026, and hope that this work will serve as a useful reference to researchers for optimizing voltammetric conditions in selecting the most effective technique.

### Different voltammetric techniques

The term “voltammetry”, introduced by Kolthoff in 1940, (ref. 46) refers to electroanalytical techniques that measure the current (ampere) moving through an electrochemical cell as a varying voltage (volt) is applied.^47,48^ Voltammetry equipment consists of electrodes, solution, and voltage control instruments. It's a system of three electrodes, *i.e.*, a working, reference, and counter electrode. The counter electrode ensures the ancillary reaction does not become rate-limiting. A steady potential is maintained by the reference electrode. And the potentiostat controls the system by regulating voltage and current distribution among the electrodes.^49,50^ With a small surface area (less than 10^−6^ m^2^), the working electrode detects the current generated by the analyte reacting to the applied potential. The electrochemical cell utilizes electrical energy to induce electrolysis, with electrodes submerged in an ionic solution containing the analyte to complete the circuit. The processes at the solution–electrode interface are critical in electroanalytical chemistry, where an external voltage or current produces an electrical response that identifies and quantifies the analyte.^46^ The applied voltage induces a variation in the amount of an electroactive substance at the edge of the electrode by electrochemically reducing or oxidizing.^49^ This article describes different types of voltammetry, which are cyclic voltammetry (CV), square wave voltammetry (SWV), and differential pulse voltammetry (DPV),^51^ anodic stripping voltammetry (ASV),^52^ and square wave anodic stripping voltammetry (SWASV),^53^ differential pulse anodic stripping voltammetry (DPASV).^54^ These methods vary in the temporal waveforms generated by their specific applications.^48^

### Differential pulse anodic stripping voltammetry

In 1976, Franke *et al.* selected DPASV owing to its capability to screen a diverse array of metals, the low LOD (parts per billion range), and the total output that can be produced, both qualitatively and quantitatively. The equipment for DPASV is less expensive than the typical AAS, and the process is comparatively straightforward.^55^ The operational expenditures are minimal, and structural necessities are limited. This approach was evaluated as an effective substitute.^56^

**Fig. 1:**
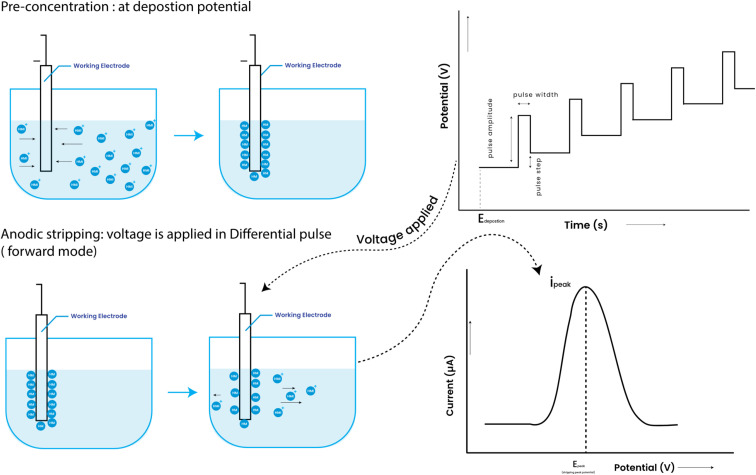
Differential pulse anodic stripping voltammetry.

Generally, DPASV (Fig. 1) is a hybrid technique that integrates differential pulse voltammetry with anodic stripping voltammetry.^57^ This technique, from the accumulation step to the initiation of the stripping part, remains the same as discussed in the anodic stripping voltammetry section.^157–162^ However, when coupled with the differential pulse technique, the voltage is applied in pulses as a temporal function rather than linear scanning. DPV's waveform is a succession of pulses with a constant baseline maintained for a specified duration prior to each potential pulse.^58^ After deposition, in the stripping step, a sequence of potential pulses, each characterized by a tiny amplitude (10–100 mV), is overlaid on a gradually varying baseline potential. Current is assessed at two intervals for each pulse: the initial point (1) immediately prior to the pulse application and the subsequent point (2) at the conclusion of the pulse. That permits the charging current to dissipate. DP differs from a regular pulse because the pulse amplitude increases in NP.^59–61^ The experimental parameters in this setting are pulse amplitude (potential difference between the baseline and the pulse amplitude), pulse width (duration of each pulse), step potential (incremental voltage change applied between each pulse), and other parameters are the same as before.^62^

Lieu *et al.* used DPASV for examining real water samples of industrial wastewater and river water. For this analysis, they optimized the operational parameters, *i.e.*, 60 mV pulse height and 8 mV step voltage, which yielded a LOD of 0.408 µg L^−1^ and 0.453 µg L^−1^ for lead and cadmium ions, respectively.^63^ Kumar *et al.* quantified the content and retention time of Lead (Pb), Cu, Zn, and Cd in air particulates, children's blood, and diet at −1.2 V (accumulation voltage) and 300 s (accumulation time) with HMDE as the working electrode from different locations in Tirupati, India.^64^ In the quantitative and multiplexed detection of Cd, Cu, and Pb ions. DPASV related data are registered in Table 1. This technique yielded very low LODs of 0.17, 0.18, and 0.69 µg L^−1^ for cadmium(ii), Pb(ii), and Cu(ii) ions, respectively.^65^ Ghoneim *et al.*, in their work on the concurrent analysis of eleven elements in water specimens with HDME, Zn was later quantified by DPASV after adjusting the pH to 4. The pH of the medium was elevated to 8.5 by the addition of NH_3_/NH_4_Cl buffer to ascertain manganese *via* DPASV. As for Cd, Cu, Sb, Pb, and Bi, 0.1 mol L^−1^ hydrochloric solution at pH (1) containing 2 mol per liter NaCl was used.^66^ In Iran, thallium ion was determined by this method using MWCNTs/L2/RTIL-CPE, with a linear range and limit of detection of 0.3–70 ppb and 0.08 ppb, respectively. When applied to actual specimens, *i.e.*, tap, lake, and river water, the percentage recovery (%) recorded was 102.5, 97.5, and 95.0, respectively. The concentration of thallium was 4 µg L^−1^ in the sample.^67^ To obtain the best result, Dong *et al.* optimized the experimental parameter using 1 µM Pb(ii) analyte. The optimal conditions recorded are pH 3.5, preconcentration voltage −1.20 V, and deposition time 120 s. Using these conditions, they analyzed water samples from different locations (tap water, Yushan Lake, Lian Lake); each sample contained 40 nM analyte, and the analysis yielded 38.4 nM, 41.3 nM, and 38.6 nM, respectively.^68^ Kinard *et al.* employed DPASV to simultaneously determine trace quantities of Zn, Cd, Pb, Cu, and Bi in industrial and domestic effluents. Fifteen distinct types of effluents from different industrial processes were used.^69^ Utilizing a new GCE modified by bismuth oxycarbide in DPASV under ideal conditions, Zhang *et al.* detected trace lead and cadmium at various quantities of 10, 20, 30, 40, and 50 ppb. ABS (pH 5) was put into action with a pulse of 25 mV height, a preconcentration voltage of −1.3 V, and a step potential of 5 mV, a *t*_d_ of 700 s.^70^ Trace metals with the LOD of 0.45 (Pb), 0.20 (Cu), and 0.10 (Cd) ppb, using Mo_6_S_9_-xIx nanowires GCE, were recorded. All the experiments in this were carried out with a pulse height of 50 mV, a period of 0.2 s, and a width of 20 × 10^−2^ s.^71^ Cu, Cd, and Pb were simultaneously measured in tap water. The specimen prepared for analysis contained 0.30 ppb, 1.50 ppb, and 5.0 ppb of each metal. And percentage recovery were found to be 103.3 for 0.3 ppb Cd(ii), 93.33 for 0.3 ppb Pb(ii), 96.66 for 0.3 ppb Cu(ii), 996.67 for 1.5 ppb Cd(ii), 95.33 for 1.5 ppb Pb(ii), 96.0 for 5.0 ppb Cd(ii), 97.6 for 5.0 ppb Pb(ii), 101.33 for 1.5 ppb Cu(ii), 96.8 for 5.0 ppb Cu(ii).^72^ Pb was determined by Dong *et al.* with a LOD of 0.02072 µg L^−1^. They used a GNP/PANI/GR/GCE in that work. The technique was applicable in the quantification of trace Pb ions in real aquatic bodies with an RSD of less than 3.5% and recovery rates of 99% (ref. 73). The DPASV method utilized a BioExt/MWCNTs/GCE sensor to detect cadmium ions, which was evaluated in two samples. While the river water underwent filtration three times and was subsequently blended with PBS at a pH value of 4.5 in a 1 : 2 volume ratio, the drinking water was analyzed directly. DPASV voltammograms were obtained under optimum conditions.^74^ Water samples from Tap Water in Canakkale (Turkey) were analyzed for Zn by DPASV. The concentration of zinc heavy metal was analyzed using a 0.2 M acetate buffer. The experimental parameters were: deposition potential −1.50 V, preconcentration time 300 s, pulse height 50 mV, and sweep rate 0.015 V S^−1^.^75^ In a work of Xiao *et al.*, the quantification of Lead and Cd was executed by DPASV at optimal conditions; deposition potential was selected as −1.2 V, a *t*_d_ of 150 s, and the supporting electrolyte at pH 4.5. Employing nitrogen-doped microporous carbon/Nafion/bismuth-film electrode.^76^ Cd(ii) in water spinach using DPASV was detected, and it was found that water spinach had a cadmium(ii) content of 0.2399 µg g^−1^. The detection process yielded an LOD of 47.28–77.1 µg L^−1^ and an *R*^2^ of 0.996. As the supporting electrolyte, 0.1 M KCl was used throughout the course of the experiment.^77^ Barceló-Quintal *et al.* implemented this technique for analyzing airborne heavy metals. They optimized the experimental conditions, focusing on cadmium, lead, zinc, and copper metal ions in urban aerosols. An HDME with a pulse height of 50 mV and sweeping at 10 mV s^−1^ was applied.^78^ With the LOD of 100 ppb for Cd, 10 ppb for lead, and 50 ppb for copper, Carrégalo *et al.* determined the concentration of the mentioned metals at pH 1 while oxygen was present. The pulse was applied in the range of −0.7 V to – 0.1 V.^79^ DPASV was utilised to find heavy metals in samples such as cereals and cereal products, vegetables, rice, fruits, animal-derived foods, mushrooms, baby food, tobacco, cigarettes, coffee, and tea, as described by Ostapczuk *et al.*, while determining heavy metals in wheat bran with a sample weight of 0.0582 g, a pulse height of 25 mV and scan rate of 5 mV s^−1^ was set. For deposition times, Zn was measured for 20 seconds at *E*_d_ = 1.2 V, Pb, Cd, and Cu for 180 seconds at *E*_d_ = −0.8 V, and Ni for 60 seconds at *E*_d_ = −0.7 V.^80^ Oyagi *et al.* successfully employed DPASV to determine Pb, Co, and Cd ions in the tap water of Nairobi City (Kenya). The optimal conditions were accumulation voltage: −0.8 V, deposition time: 300 seconds, pulse height: 0.06 V, pulse period: 0.02 seconds, and sampling width: 0.0033 seconds. The LODs of Co(ii), Pb(ii), and Cd(ii) were approximately 0.9 × 10^−9^ M, 1.9 × 10^−9^ M, and 11 × 10^−6^ M, respectively, and were measured.^81^ DPASV was utilized and optimized to enhance its sensitivity for determining As(III), as arsenic(v) is not electrolytically reducible in most supporting electrolytes. Therefore, to reduce As(v) to As(iii), Na_2_S0_3_ was used as the reductant. Under optimal conditions, an RSD of 8.4% and 5.4% were recorded for a *t*_d_ of 5 minutes and 1 minute, respectively.^82^ Copper, cadmium, lead, and zinc were determined with DPASV in wine. In this, using the traditional addition technique, the exactitude of the results was evaluated, and the recoveries varied from 82.5 to 130.8% for zinc, from 89.7 to 101.0% for lead, and from 81.4 to 105.9% for copper, as well as from 85.7 to 107.0% for cadmium.^83^ Arsenic in mine-influenced waters was investigated using this method. Samples of water were taken from Roodepoort, Johannesburg, which is an old gold mining area with a number of mine dumps. The process for using thiosulfate to chemically reduce As(v) to As(iii) before DPASV measurements was optimized.^84^

1 2H_3_AsO_4(aq)_ + 2H^+^ + S_2_O_3_^2−^ → 2H_3_AsO_3(aq)_ + SO_2_ + H_2_0

2 2H^+^ + S_2_O_3_^2−^ → S + SO_2_ + H_2_0

An investigation into Zn(ii), Cu(ii), Pb(ii), and Cd(ii) during the refinement of various vegetable oils was conducted by using DPASV. The effectiveness of the removal of Zn(ii), Cu(ii), Pb(ii), and Cd(ii) during refinement was measured to be 98.50–99.90%, 98.20–99.91%, 95.26–95.93–99.92%, and 99.76%, respectively, with *R*^2^ of 0.9930, 0.9928, 0.9893, and 0.9931 (ref. 85). Yan *et al.* investigated optimal conditions to determine Pb(ii) using a GCE coated with *N*-doped carbon-modified palygorskite. The following experimental conditions yielded the best results: (b) deposition time: 180 s; (a) accumulation voltage: 1.2 V; (c) pH: 4.5 (ref. 86). Metal ions in contaminated water were analyzed with boron-doped nanocrystalline diamond thin-film electrodes by DPASV. The concentrations were determined by DPASV, which is (124 ± 5) µg L^−1^ for Pb and (156 ± 5) µg L^−1^ for Cd, with coefficients of variance of 4.0% and 3.2% at a pH value of 4.5 (0.1 M ABS).^87^ Ions of Cd and Pb in ecological specimens were measured using polyrutin/silver nanoparticles on GCE. Pb and Cd ions exhibited linear concentrations between 6.01 µg L^−1^ and 29.01 µg L^−1^, and 6.41 µg L^−1^ to 22.82 µg L^−1^, respectively, with LOD of 0.62 µg L^−1^ and 1.12 µg L^−1^.^88^ Yoo *et al.* reported a way to determine trace Hg by DPASV with a correlation factor of 0.9995 using a polythiophene-quinoline modified and 0.1 M KCl (pH 3).^89^ Yansheng *et al.* analyzed Cd(ii) with an exceptionally low 1.2 µg L^−1^ limit of detection and a wide 5–100 µg L^−1^ dynamic linear range. The sensor demonstrated commendable accuracy (96–103% recovery) in detecting Cd(ii) from spiked industrial effluent and tap water samples in 2024. In that study a novel imprinted polyaniline-gold nanoparticle (PANI-AuNPs) nanocomposite modified glassy carbon electrode was strategically constructed and employed under optimum conditions *i.e.* 180 s quiet time accumulation at −1.2 V with modulation ampli-tude of 25 mV, pulse width 50 ms, pulse period 500 ms in acetate buffer (0.1 M, pH 5) and demonstrated a <5% relative standard deviation reflecting the sensor's accuracy and precision.^90^ In 2026, Cd(ii) was investigated using Ni-Based Metal–Organic Framework as an electrochemical sensor through the optimization of DPASV, which demonstrated a superior sensitivity of 2.95 × 10^−5^ A ppm^−1^ and linear range of 0.01 to 7000 µg L^−1^.^91^

**Table 1:** Optimized experimental parameters and analytical performance for the detection of heavy metal ions by the DPASV technique

Heavy metal	Pre-concentration potential (V)	Deposition time (s)	Pulse amplitude (mV), scan rate (mV s^−1^)	Peak potential (V)	Limit of detection (LOD) (µg L^−1^)	Working electrode	Electrolyte & pH	Ref.
Cd(ii)	−1.1	120	60, 0.25	—	0.453	(PtNFs/GCE)	0.10 M ABS & pH 4.5	63
Pb(ii)					0.408			
Cd(ii)	−1.1	500	50, 50	−0.62	0.17	(Hg/rGO)_film_/GCE (GCE with a mercury-on -graphene film)	0.010 M HNO3 buffer solution containing 0.02 M KNO_3_ & –	65
Cu(ii)				+0.02	0.69			
Pb(ii)				−0.45	0.18			
Cd(ii)	−0.95	30	25, 2–4	—	0.015	Hanging mercury drop electrode (HMDE)	0.10 M HCl containing 0.2 M NaCl & pH 1	66
Pb(ii)					0.023			
Sb(iii)			25, 2		0.016			
Cu(ii)					0.012			
Bi(iii)					0.038			
Zn(ii)	−1.28		25, 4		0.013		0.1 M HCl containing 0.2M NaCl + NH_3_/NH_4_Cl buffer & pH 4	
Mn(ii)	−1.72		25, 2 or 4		0.046		0.10 M HCl containing 0.2 M NaCl + NH_3_/NH_4_Cl buffer & pH 8.5	
Tl(i)	−1.1	180	50, –	—	0.08	MWCNTs/L_2_/RTIL-CPE	0.1 M PBS & pH 6	67
Pb(ii)	−1.20	120	50, –	—	0.207	PANI/MMT/GCE	0.2 M ABS & pH 3.5	68
Pb(ii)	−1.3	700	25, –	−0.56	4.30	Bismuth oxycarbide modified GCE	Hac-NaAc solution & pH 4.5	70
Cd(ii)				−0.81	3.97			
Cd(ii)	−1.1	240	50, –	—	0.10	Mo_6_S_9−*x*_I_*x*_ nanowires modified glassy carbon electrode (GCE)	0.1 M HAc-NaAc solution & pH 4.7	71
Cu(ii)					0.20			
Pb(ii)					0.45			
Cd(ii)	−1.2	180	50, –	−0.82	0.02	RGO-CS/PLL/GCE	0.1 M acetate buffer & pH 4.5	72
Pb(ii)				−0.54	0.02			
Cu(II)				−0.11	0.02			
Pb(ii)	−1.2	240	50, -	—	0.0207	Gold nanoparticle/polyaniline/graphene ternary nanocomposite modified GCE (GNP/PANI/GR/GCE)	0.1 M ABS & pH 6.0	73
Cd(ii)	−1.5	120	50, 50	—	0.1135	GCE modified with BioExt/MWCNTs nanobiocomposite (BioExt/MWCNTs/GCE)	0.1 M PBS &pH 4.5	74
Zn(ii)	−1.5	300	50, 15	−0.98	—	Glassy carbon electrode (GCE)	0.2 M ABS & pH 3.5	75
Cd(ii)	−1.2	150	50, –	−0.84	1.5	(Nafion/Bi/NMC/GCE)	0.1 M ABS & pH 4.5	76
Pb(ii)				−0.60	0.05			
Cd(ii)	−1.1	360	–, 100	—	47.28–77.1	Glassy carbon electrode	0.1 M KCl & -	77
Pb(ii)	−1.2	90	50, 10	—	0.8	HMDE	Nitric acid & –	78
Cd(ii)					0.05			
Cu(ii)					0.29			
Zn(ii)					0.26			
Cd(ii)	−1.4	60	—	—	10	Graphite-epoxy composite electrodes (GEC)	0.1 M HCl & pH 1	79
Pb(ii)					10			
Cu(ii)					50			
Pb(ii)	−0.8	300	60, –	−0.48	0.186	Polyacrylic acid/GCE	Acetate buffer & pH 6.0	81
Co(ii)				−1.19	648.26			
Cd(ii)				−0.745	0.101			
As(iii)	−0.2	60	50, 10	—	0.02	Hanging mercury drop electrode (HDME)	1 M HClO_4_ & –	82
Zn(ii)	−1.1	120	50, 20	—	—	HMDE	0.1M ammonium acetate solution & pH 4.6	83
Cd(ii)								
Pb(ii)								
Cu(ii)								
As(iii)	−0.4	120	–, 200	—	—	Solid gold electrode	0.5 M HCl & –	84
Cd(ii)	−1.2	300	50, 20	—	0.097	HMDE	0.2 M KNO_3_	85
Zn(ii)					1.37			
Cu(ii)					0.216			
Pb(ii)					1.47			
Pb(ii)	−1.2	180	25, –	—	0.42	PAL/C–N/GCE (*N*-doped carbon-modified palygorskite coated GCE)	0.10 M ABS & pH 4.5	86
Cd(ii)	−1.0	180	50, –	−0.75	—	Boron-doped nanocrystalline diamond thin-film electrode	0.1 M Hac-NaAc & pH 4.5	87
Pb(ii)				−0.51				
Cu(ii)	−1.25	180	–, 30	0.054	39	Glassy carbon electrode	0.1 M pyrophosphate & pH 4	92
Cd(ii)				−0.804	9.6			
Zn(ii)				−1.017	14.7			
Pb(ii)				−0.049	16.6			
Cd(ii)	−1.4	270	—	−0.79	0.86	Stannum–bismuth composite film electrode	0.1 M Hac-NaAc & pH 4.7	93
Zn(ii)				−1.15	0.31			
Zn(ii)	−1.15	1800	50, 8	—	0.05	Hanging mercury drop electrode	MnSO_4_ & pH 4.1	94
Cd(ii)					0.03			
Pb(ii)					0.10			
Cr(iii)	−1.4	180	50, 20	−1.18	2.0	Stannum film electrode	0.1 M Hac-NaAc & pH 5.3	95
Cd(ii)				−0.84	1.1			
Pb(ii)	−1.0	120	50, 20	−0.41	1	Hanging mercury drop electrode	—	96
Cd(ii)				−0.61	1			
Pb(ii)	−1.3	330	50, 0.1	—	0.7	*N*-doped nanoporous carbon-casted GCE	0.1 M acetate buffer & pH 5	97
Pb(ii)	−1.0	120	100, 50	−0.46	—	Nitrogen-doped diamond-like carbon film electrode	0.1 M KCl & pH 1	98
Cd(ii)				−0.63				
Cd(ii)	1.2	120	80, 50	—	0.09	(BiF/GNFs-NA/GCE)	0.10 M Hac-NaAc solution & pH 4.5	99
Pb(ii)					0.02			
Cu(ii)	−1.0	300	50, 15	—	1.1	Calix^4^ arene modified CPE	0.1 M HCl & pH 6.5–7.5	100
Pb(ii)	−1.2	600	80, 50	−0.54	0.001	(GR-AuNPs-CS/GCE)	0.10 M ABS & pH 4.5	101
Cd(ii)	−1.4	60	—	—	100	Graphite-epoxy composite electrodes	0.1 M HCl & pH 1	102
Pb(ii)					10			
Cu(ii)					50			
Hg(ii)	−0.2	180	–, 50	—	0.00005	Poly (methane disulfide)/Au nanoparticle/MWCNT modified glassy carbon electrode	0.1 M KCl & -	103
Zn(ii)	−1.5	120	50	—	3.5	Bismuth film electrode (BiSPCE)	0.1 M KNO_3_ and maleic/maleate buffer & pH 6	104
Pb(ii)					0.5			
Cd(ii)					3.9			
Pb(ii)	−1.0	240	– & 50	—	1.6	Poly xylenol orange modified electrode	0.1 M Hac-NaAc & pH 5	105
Cd(ii)	−1.2	180	25, –	−0.55	1.2	PANI-AuNPs/GCE	0.1 M acetate buffer & pH 5	106

### Square wave anodic stripping voltammetry

**Fig. 2:**
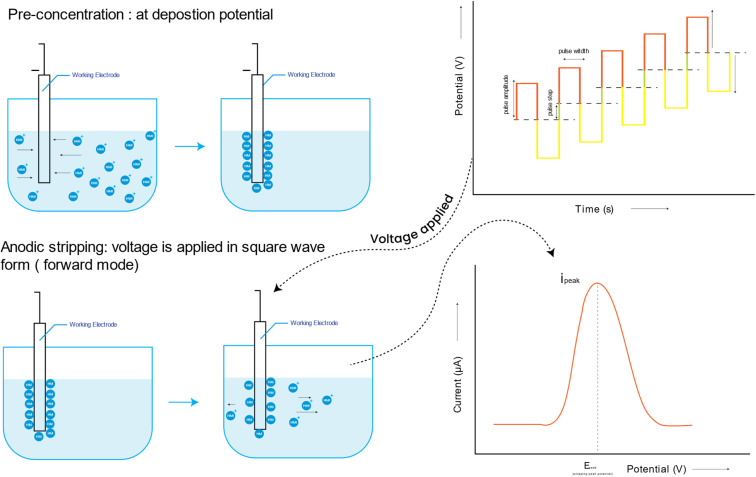
Square wave anodic stripping voltammetry.

In a recent study published in 2024 by Deffo *et al.*, it was stated that SWASV (Fig. 2) is one of the most sensitive electroanalysis techniques^107^ It is a commonly used method among the electrochemical detection techniques.^108^ The use of SWASV for trace-element analysis has recently grown in popularity. SWASV can perform scans at faster rates than other voltammetric methods and can analyze numerous elements for a single sample in a manner comparable to other voltammetric methods,^109^ It exhibits notable selective ability and detection capacity (approximately 10–12 mol L^−1^), is economically feasible relative to spectroscopic and chromatographic practices, and is extensively employed for trace metal study and more than one element assessment.^110^ SWASV related data are registered in Table 2.

**Table 2:** Optimized experimental parameters and analytical performance for the detection of heavy metal ions by the SWASV technique

Heavy metal	Pre-concentration potential (V)	Deposition time (s)	Pulse amplitude (mV), frequency (Hz)	Peak potential (V)	Limit of detection (LOD) (µg L^−1^)	Working electrode	Electrolyte & pH	Ref.
Cd(ii)	−1.2	180	25, –	−0.87	0.15	Bismuth nano-powder electrode	0.1 M NaAc and 0.025 M HCl solution & pH 5.0	122
Zn(ii)				−0.63	0.07			
Pb(ii)	−1.3	120	25, 25	—	3.53	3D-printed metal electrode	0.1 M ABS & pH 4.5	123
Cd(ii)					9.53			
Cd(ii)	−1.2	240	20, 25	—	—	Antimony-hydroxyapatite modified electrodes (Sb-HAp-CME)	0.1 M HCl & pH 2	125
Pb(ii)								
Cd(ii)	−1.2	30	25, 20	−0.65	1.3	Novel modified-NPBiE	0.1M acetate buffer solution & pH 4.6	127
Pb(ii)				−0.45	1.5			
Cd(ii)	−1.1	40	—	—	0.089	Green synthesized silver nanoparticle-modified carbon paste electrode	0.1M ABS & pH 6	128
Pb(ii)					48			
Pb(ii)	—	600	50, 10	−0.641	0.207	Nafion/CNTs/Benzo-18-crown-6 modified GCE	0.1 M ABS & pH 4.5	129
Pb(ii)	−1.0	180	35, 25	—	1.13	Bismuth film electrode (BiFE)	30 mM oxalic acid and 150 M CTAB & pH 4	116
Sn(iv)					0.74			
Cd(ii)	−1.2	300	25, 25	−0.7	1.7	Sb_2_O_3_ modified carbon nanotube paste electrode	0.01 M HCl & pH 2	117
Pb(ii)				−0.5	1.2			
Hg(ii)	−1.0	300	25, 15	+0.3	5.616	Stainless steel electrode (Type 304)	0.1 M acetate buffer & pH 4.5	119
Pb(ii)				−0.41	6.837			
Cu(ii)				−0.05	0.463			
Cd(ii)				−0.7	25.855			
Hg(ii)	−1.15	180	110, 50	+0.283	0.06	L/CuO–CoO–MnO/SiO_2_/IL modified CPE	BR buffer & pH 3.5	118
Cd(ii)				−0.832	0.0387			
Pb(ii)				−0.424	0.0498			
Zn(ii)				−1.1	0.033			
Pb(ii)	−1.2	600	50, 50	—	0.05	PPy and CNFs modified CPE	0.1 M acetate buffer & pH 4.5	133
Cd(ii)	—	150	25, 25	—	0.6	rGO/CNT modified BiE	0.1 M acetate buffer & pH 4.5	134
Pb(ii)					0.2			
Cd(ii)	−1.2	120	25, 25	—	0.77	Antimony nanoparticle-multiwalled carbon nanotubes composite immobilized at carbon paste electrode	0.01 M HCl & pH 2	135
Pb(ii)					0.65			
Zn(ii)	−1.3	140	—	—	0.00058	Amino acid-fabricated glassy carbon electrode (Ala/GCE)	BR buffer & pH 4	130
Cu(ii)					0.00019			
Cd(ii)					0.00065			
Hg(ii)					0.00118			
Pb(ii)	−1.4	150	25, 25	—	0.4	TRGO/Au electrode (micro-patterned reduced graphene Oxide)	0.1 M ABS & pH 4.5	131
Cd(ii)					1.0			
Cd(ii)	−0.7	150	5, –	−0.76	2.6	Amino functionalized Fe_3_O_4_@Carbon microspheres modified electrode	0.1 M Hac-NaAc & pH 5	136
Cu(ii)				0 V	2.44			
Pb(ii)				−0.52	5.905			
Cd(ii)	−1.2	120	25, 25	−0.84	0.12	Bismuth modified hybrid binder carbon paste electrode	0.11 M acetate buffer & pH 4.5	141
Pb(ii)				−0.58	0.25			
Cd(ii)	−1.2	240	50, 50	—	0.099	Novel nanocomposite of Bi-PPy/MWCNT modified CPE	0.1 M acetate buffer solution & pH 4.5	142
Pb(ii)					0.157			
Cd(ii)	−1.0	180	50, 25	−0.75	0.90	IONP-COOH/APTES-ITO-modified electrode	1 M acetate buffer & pH 4.5	143
Pb(ii)				−0.48	0.60			
Cd(ii)	−1.4	120	40, 50	—	0.7	Tin-film electrode	1 M acetate buffer & pH 4.5	144
Zn(ii)					1.1			
Zn(ii)	−1.2	50	25, –	—	0.004	Bis-triazole-based calix^4^ arene-modified GCE	BRB solution & pH 3	145
Pb(ii)					0.003			
As(iii)					0.005			
Hg(ii)					0.005			
Ni(ii)	−1.0	180 s	25, 25	−0.325	—	GCE	0.1 M ammonium chloride solution & pH 4.5	146
Cd(ii)	−1.0	135	20, 20	−0.84	0.487	*N*-[(Benzyloxy)carbonyl]-L-alanyl-L-prolyl-l-leucine-*N*-cyclohexylcyclohexanamine (Cbz-APL) tripeptide-coated GCE	Britton-Robinson buffer & pH 5	147
Hg(ii)	−1.0	150 s	25, 15	—	1.79	SN-rGO/GCE	0.1 M ABS & pH 5	148
Pb(ii)	−1.0	350	5, –	—	5	ZIF-67/rGO	Acetate buffer solution & pH 5	149
Cd(ii)	−1.0	400			2.93			
Hg(ii)	−0.8	180	45, –	0.166	0.323	Thiourea grafted Chitosan modified carbon paste electrode (TU-gt-Cts/CPE)	—	150
Cd(ii)	−1.2	120	25, 50	—	0.08	Bi/GR/IL composite modified SPE	0.1 M ABS & pH 4.5	151
Pb(ii)					0.10			
Cd(ii)	−1.4	180	25, 25	—	3.55	Metal-bismuth modified pre-anodized screen-printed electrode	0.1 M ABS & pH 4.5	152
Cd(ii)	−1.2	300	—	−0.70	0.03	Zirconium oxide nanoparticles and MWCNTs/CPE	0.1 M ABS & pH 5	153
Pb(ii)				−0.46	0.049			
Hg(ii)				+0.20	0.082			
Cd(ii)	−0.9	210	100, 50	—	6.6	Phosphorus yilde *N*-BDMP/CPE	BRB solution & pH 3	154
Hg(ii)					8.2			
Pb(ii)	−1.0	120	–, 25	−0.05	11.81	Nanoporous gold microelectrode (NPG-µE)	0.1 M ABS & pH 4	155
Zn(ii)	−1.3	420	25, 15	−1.15	0.52	Bi@In hybrid nanofilm on glassy carbon electrode	0.1 M ABS containing 0.1 M KCl & pH 6.0	156
Cd(ii)				−0.82	0.15			
Pb(ii)				−0.58	0.67			
Zn(ii)	−1.2	180	50, 25	−1.0	0.48	TbFeO_3_/CuO/CPE	ABS & pH 4.8	157
Cd(ii)				−0.73	0.29			
Pb(ii)				−0.51	0.12			
Cd(ii)	−1.0	0.2	25, 15	−0.73	309.13	BiVO_4_/GCE	0.1 M HEPES buffer and pH 8.0	158
Pb(ii)				−0.53	480.70			
Cu(ii)				−0.07	172.86			
Hg(ii)				0.22	240.71			
Cd(ii)	−1	240	25, 25		1.80	PWA@β-CD-based NSs/MWCNTs/CPE	0.1 M acetate buffer & 4.5	159
Pb(ii)					2.90			
Pb(ii)	−1.2	450	25, 25	−0.6	1.21	PAMAM/Ni-MOFs/GCE	Acetate buffer & pH 5	160
Cu(ii)				−0.05	0.77			
Cd(ii)	−0.8	30	25, 25	−0.8	7.87	Mn_2_O_3_ NPs/CPE	PBS & pH 3.5	161
Pb(ii)				−0.55	39.37			
Cd(ii)	−1.0	300	0.08		3.37	NH_2_-MIL-53(Fe)/GCE	Acetate buffer solution (ABS) pH 5.5	162
Cd(ii)		270	100, 80	−0.768	0.003	HDPBA–CPE	Britton–Robinson (B–R) buffer (0.1M, pH 4)	163
Zn(ii)		120	60, 40	−1.190	0.162	HDPBA–MWCNTs/CPE	0.1 M Brinton–Robinson (B–R) buffer solution (pH 6)	164
Hg(ii)	−0.8	210	75, 50	0.187	0.256	HDPBA–CPE	0.1 M NH_4_ Cl solution (pH 4)	165
Pb(ii)	−0.9	270	80, 60	−0.594	0.002	HDPBA–CPE	0.1 M sodium acetate (NaAc) pH 5	166
Cu(ii)	−0.70	180	100, 60	−0.030	0.0003	HDPBA–MWCNTs/CPE	0.1 M ammonium chloride (NH_4_Cl, pH 5)	167

This technique is used in electrochemistry to amplify small peak currents generated by trace amounts of analytes. It combines standard voltammetric techniques with a preconcentration phase, where the sample is electrochemically deposited upon the exterior of the electrode, increasing its effective absorption and enabling its detection.^111^ Combining the SWV with ASV techniques is the usual way to do SWASV.^56^ In this technique, the process from the preconcentration step to the initiation of the stripping part remains the same, as discussed in the anodic stripping voltammetry part.^157–162^ However, voltage is applied in a square waveform from the stripping step. That is, the working electrode's potential changes through a sequence of positive and negative pulses from a starting potential to a final one. The square amplitude determines the forward step, whereas the square increment subtracts it from the square amplitude to calculate the backward step.^112^ The process is divided into two phases. The first is called preconcentration, and it involves applying either forward or backward potential to the electrodeposition of MIs in solution, which causes the MIs to accumulate as amalgam. The second stage causes the metal in the mercury electrode to oxidize by sweeping the *E*_WE_ to reversal potentials. When the SW potential is applied to the WE, electric currents are generated. Every square-wave cycle has two measurements of the current: one at the conclusion of the forward pulse (*i*_f_) and one at the conclusion of the reverse pulse (*i*_r_). The base staircase potential is plotted against the variance between both observed currents (Δ*i* = *i*_f_ − *i*_r_), yielding a peak-shaped voltammogram that exhibits symmetry over the half-wave potential, with *i*_peak_ current being precisely correlated to the concentration.^113^ The following experimental conditions and parameters are involved in SWASV: pH, supporting electrolyte, scan rate, preconcentration potential, preconcentration time, scanning potential range, square wave amplitude, frequency, step potential, and equilibration time.^114^

With a recovery value between 103.7 and 108.4% and with a LOD of 0.0574 µg L^−1^, Berrabah *et al.* detected Cu(ii) with SWASV. The concentration range was between 10.00 × 10^−9^ M and 6.00 × 10^−7^ M (ref. 115). Tang *et al.* investigated and optimized the experimental conditions to determine Lead and Tin simultaneously. Thirty Millimoles per liter oxalic acid and 150 micromoles per liter CTAB were picked as the optimized supporting electrolyte, with 25 Hz and 4 mV identified as the best possible potential step and frequency, respectively.^116^

Under ideal conditions, Cd(ii) and Pb(ii) were identified with a linear concentration dependency ranging from 10 to 100 µg L^−1^ using an Sb_2_O_3_/CNTCPE electrode. The concentrations of Pb(ii) and Cd(ii) were both 20 µg L^−1^.^117^ In the investigation of Pb(ii), Hg(ii), Zn(ii), and Cd(ii) by Faridan *et al.*, the highest oxidation peak was obtained using BRB solution as supporting electrolyte (pH 3.5), deposition voltage −1.15 V, preconcentration time 180 s, step voltage 5 mV, Pulse height 110 × 10^−3^ V.^118^ A Type 304 stainless steel WE was put in action by Kitte *et al.* in their study on Cu, Pb, Hg, and Cd ions. And the method best operated at +0.3 V for Hg(ii), −0.05 V for Cu^2+^, 0.41 V for Pb(ii), and −0.7 V for Cd^2+^ after depositing for 300 s at −1.0 V.^119^ The concentrations of lead and cadmium in canned food had been evaluated within a range of 50 to 150 µg L^−1^. A regression coefficient of 0.999 for both was recorded using this technique.^120^ Li *et al.* report a determination method for Pb(ii) using SWASV, carried out at *E*_d_ = −0.90 V, *t*_d_ of 4 min, and acetylene black (AB) paste electrode as working electrode with 0.1 mol L^−1^ HClO_4_ solution comprising 7.0 mM KI as assessment media.^121^ Cd(ii) and Pb(ii) were examined utilizing a bismuth nanopowder electrode, achieving LOD of 0.150 µg L^−1^ for Cd ions and 0.070 µg L^−1^ for Pb ions. The supporting electrolyte was a 0.1 M ABS and 0.025 M HCl solution (at a pH value of 5). The accumulation step of Cadmium(ii) and lead(ii) proceeded for 180 s at 1.2 V while applying magnetic agitation.^122^ With a 3D-printed Metal Electrode, Lee *et al.* determined Pb and Cd with LODs of 3.53 and 9.53 ppb and regression coefficients of 0.989 and 0.964, respectively. At room temperature, measurements were performed at a pH of 4.5 in 10 mL of 0.1 M ABS with dissolved oxygen present. A SW potential scan ranging from 1.3 V to 0.3 V was executed, and the corresponding voltammogram was documented at a frequency of 25 Hz, a height of 25 mV, and a voltage step of 4 mV (ref. 123). The methodology has been developed by Silakorn *et al.* for the quantification of ultra-trace concentrations of mercury, and the experiments were carried out by setting parameters as follows: a pulse amplitude of 20 mV, a SW frequency of 25 Hz, a step height of 5 mV, and the voltage scan ranged from −0.4 V to 0.2 V at room temperature.^124^ SWASV electrochemical research has been used to more efficiently detect HMIs. The experiment was performed in 0.1 M HCl at pH 2 with five minutes of N_2_ purging and rotation speed at 600 rpm. A correlation coefficient of 0.993 was recorded.^125^ SWASV was employed to determine Pb(ii) in a sample of blood, which was collected with EDTA and diluted 20 times, and treated with 1.2 M HCl. LOD and Data linearity range was found to be 5 µg L^−1^ and 0–5 µg L^−1^, respectively.^126^ In the determination of *in situ* heavy metal, a novel modified nanoporous bismuth electrode was used. Cadmium and Lead were determined with two sharp peaks at 0.65 V and 0.45 V in a 0.1 M acetate buffer solution at pH 4.6, respectively.^127^ Cadmium ions and Lead ions were assessed in textile-discharged effluent employing SWASV. The technique displayed a very low LOD: 48 µg L^−1^ and 89.1 µg L^−1^ for lead and cadmium.^128^ Under ideal conditions, such as pH 4.5, reduction potential −1 V, the approach demonstrated a very low LOD, with deposition and reduction times of 10 minutes and 2 minutes, respectively, in a 0.1 M acetate buffer, using a Nafion/CNTs/Benzo18-crown-6 modified GCE as the WE, according to Anandhakumar *et al.*, who employed this technique to detect Lead(ii).^129^ Britton Robinson buffer of pH 4 was found to be the most suitable supporting electrolyte to detect copper, zinc, cadmium, and mercury. A detection limit of 8.92, 5.77, 3.01, and 5.89 pM was obtained for the metal ions, respectively.^130^ Xuan *et al.*, in their determination of heavy metal detection for the Pb and Cd ions, found correlation coefficients of 0.9922 and 0.9828 for the respective metal ions. The linear regression equation for Cd(ii) is established as *I*_cd_ = 0.01 + 0.045C_Cd_, demonstrating an accuracy of 0.05 ± 0.01 µA µgL^−1^. For Pb(ii), the corresponding equation is *I*_pb_ = 0.02 + 0.065C_Pb_, with an accuracy of 0.07 ± 0.01 µA µgL^−1^.^131^ Eggs of turtle and algae from Guanahacabibes Protected Sea Park were examined using SWASV by Tamayo *et al.* at a conditioning potential of −0. 3 V for 60 seconds, a deposition voltage of −1 V for 180 s, an equilibration duration of half a minute, a SW amplitude of 28 mV, a step voltage of 3 mV, and a frequency of 15 s^−1^ (ref. 132). SWASV was developed by optimizing its experimental conditions to determine trace Pb(ii). Under those conditions, three tap water samples were analyzed, and RSD (%) of 1.2 (tap water 1; concentration 5 µg L^−1^), 2.1 (tap water 2; concentration 5 µg L^−1^), and 2.3 (tap water 1; concentration 5 µg L^−1^) were measured.^133^ Xuan and Park, at *t*_dep_ = 150 s; *t*_eq_ = 20 s; *E*_begin_ = −1.4 × 10^6^ µV, *E*_end_ = −0.6 × 10^6^ µV, *E*_step_ = 5 mV, *E*_ampl_ = 25 mV, and *f* = 25 s^−1^ determined lead and cadmium with a limit of detection of 0.2 µg L^−1^ and 0.6 µg L^−1^ with this method.^134^ At pH 2, 0.01 mol L^−1^ HCl as supporting electrolyte, Lead and Cadmium were examined in the range of 10.0–60.0 µg L^−1^ by Ashrafi *et al*. Both metal ions produced linear calibration curves, yielding *R*^2^ values of 0.999 for lead and 0.998 for cadmium.^135^ Bai *et al.* used SWASV with an Electrode Modified by amino-functionalized Fe3O4@Carbon Microspheres to determine copper, cadmium, and lead separately and simultaneously. The ideal condition was found to be Hac-NaAc buffer as supporting electrolyte, pH 5, position potential 0.7 V, *t*_d_ 150 s.^136^ At ideal conditions, *i.e.*, deposition potential at −0.5 V for 100 s in 0.1 M Hac-NaAc with pH 5.0, As(iii) was determined with a LOD of 470 000 µg L^−1^.^137^ A single-walled carbon nanotubes-copper metal–organic framework for Pb(ii) exhibited a detection limit of 25 nM and sensing ability of 0.1499 µA nM^−1^.^138^ In a very recent study conducted in 2025, incorporating SWASV Cd(ii), Pb(ii), Cu(ii), and Hg(ii) were detected with low detection limits of 309.13 µg L^−1^, 480.70 µg L^−1^, 172.86 µg L^−1^, and 240.71 µg L^−1^, respectively, using bismuth vanadate (BiVO_4_) nanospheres integrated GCE and the lower RSD values obtained for Pb(ii) (2.61%) and Cd(ii) (8.45%) indicate high reproducibility, while the slightly higher RSD values for Cu(ii) (12.37%) and Hg(ii) (8.89%) suggest minor variability. A linear detection range of 0 µM to 110 µM was also obtained.^139^ In another study in the same year, PWA@β-CD-based NSS/MWCNTs/CPE under optimized conditions, *i.e.*, PWA@β-CD-based NSs 10.0% (w/w), MWCNTs 5.0% (w/w), pH 4.5, deposition potential −1.0 V, and deposition time 240 s were employed to detect Cd(ii) and Pb(ii). The sensor demonstrated excellent reproducibility, with relative standard deviation (%RSD) values below 6.2%, and delivered satisfactory recovery rates ranging from 92% to 106% in real-world environmental samples.^140^

### Anodic stripping voltammetry

Zbinden presented the first empirical use of this approach in 1931 when he attempted to measure copper electrogravimetrically by monitoring the current while stripping.^168^ Ariel and Eisner employed this in one of its initial ecological applications in 1963 to assess copper, cadmium, and zinc in Dead Sea brine.^169^ It is a cost-effective, receptive, and accurate electroanalytical method for finding minor amount of metals.^169^ It is predominantly utilized for trace metal analysis. And possesses a feasible LOD in the ppt level, which is combined with the capacity to concurrently quantify 4 to 6 trace metals, utilizing reasonably cost-effective instruments.^59,170^

Anodic stripping voltammetry, by definition, is a stripping analysis that involves plating metal ions from solution onto a working electrode over a certain amount of time, then stripping the deposited metal in a subsequent phase.^171^ Three major processes comprise electrochemical stripping analysis. (a) The electrolytic concentration of the analyte is obtained through stirring the mixture or turning the electrode using suitably repeatable equipment. This stage is known as preconcentration. (b) Equilibration or rest interval happens before stripping by turning off the deposition stirring mechanism for 15–30 seconds. (c) The deposited analyte can be electrochemically removed from the electrode through the stripping process, facilitating analytical measurement. During this step, the voltage is scanned in either direction in order to eliminate the substance of interest from the electrode.^172^

The preconcentration in anodic stripping is accomplished through controlled cathodic deposition at a potential greater than the elemental reduction potential and for a duration of time. Through processes of convection and diffusion, the MIs are carried to the exterior of the electrodes, where they undergo reduction and concentration as amalgams in the mercury. Next, the potential is linearly and anodically scanned. In a sequence that depends on the standard potential of each metal, the amalgamated metals are removed from the electrode, reoxidized, and the resulting anodic peak currents are recorded. There is a straight correlation between the C-metal ion and the peak current, *i*_p_.

In the pre-concentration step:

3 M^*n*+^ + *n*e^−^ → M^0^; mercury free electrode

4 M^*n*+^ + *n*e^−^ → M(Hg); mercury electrode

Stripping step:

5 M^0^ − *n*e^−^ → M^*n*+^; mercury free electrode

6 M(Hg) − *n*e^−^ → M^*n*+^; mercury electrode

**Fig. 3:**
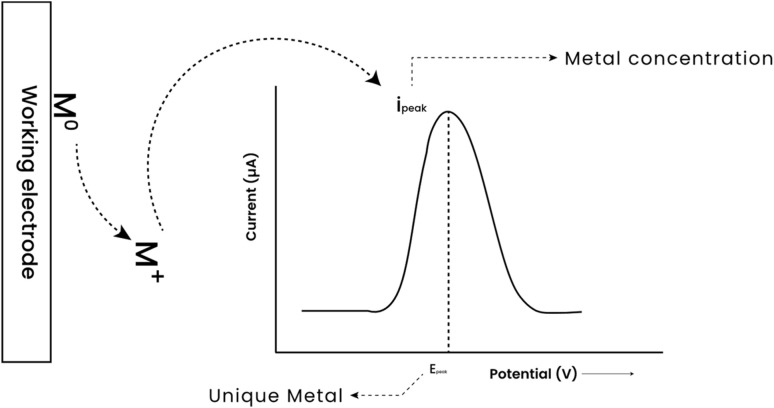
Metal stripped out of the electrode, reoxidized, and gives rise to anodic peak currents.

**Fig. 4:**
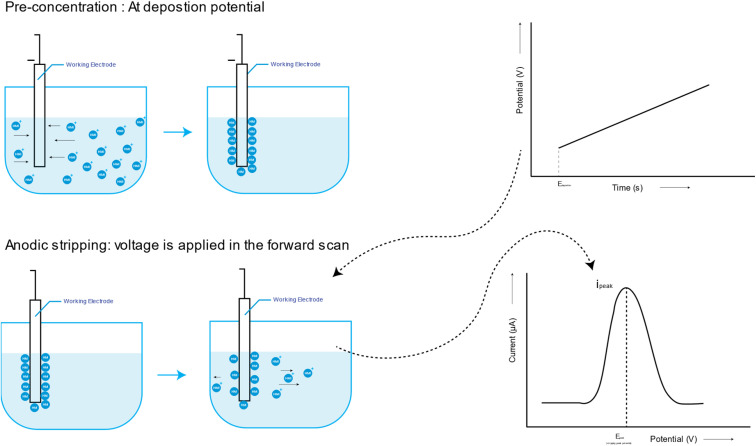
Anodic stripping voltammetry in linear mode.

Approximately 20 amalgam-producing metals can be quantified by ASV (related data are given in Table 3) using HgE, including cadmium, lead, copper, zinc, indium, bismuth, thallium, antimony, tin, nickel, cobalt, and gallium.^169,172–174^ For (HMDE), the stripping peak current can be calculated as
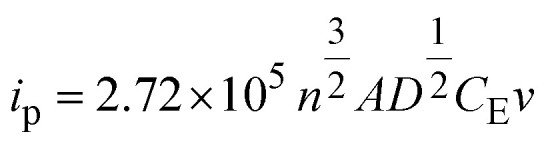
where *C*_E_ represents the concentration of the substance of interest in Hg drop, and *v* denotes the rate of ramping for the voltage scan.^175^ Instrumentation in ASV (Fig. 3 and 4) is similar to cyclic voltammetry. However, as WE, HMDE is widely used, which is set in a way that it can rotate, or some other instrumentation is used to stir the solution.^176^

**Table 3:** Optimized experimental parameters and analytical performance for detecting heavy metal ions by the ASV technique

Heavy metal	Pre-concentration potential (V)	Deposition time (s)	Scan rate (mV s^−1^)	Peak potential (V)	Limit of detection (LOD) (µg L^−1^)	Working electrode	Electrolyte& pH	Ref.
Cu(ii)	−1.5	120	100	0.024	0.017	CNT thread electrode	0.1 M ABS & pH 4.5	178
Pb(ii)				−0.488	0.311			
Cd(ii)				−0.716	0.21			
Zn(ii)				−0.980	0.09			
Cd(ii)	−1.2	120	—	−0.767	—	Antimony film carbon paste electrode	0.01 M hydrochloric acid & pH 2	179
Pb(ii)				−0.520				
Pb(ii)	−0.70	120	—	−0.44	103.6	Mercury microelectrodes	0.1 M KNO_3_	180
Cd(ii)	−0.90	120		−0.61	56.2			
Hg(ii)	−1.1	20	—	+0.30	220.649	CPE in the presence of PEI	0.05–0.1M KNO_3_ & pH 2–3	182
Ag(i)	−0.5	60		+0.28	92.76			
Cu(ii)	−0.8	40		−0.05	114.38			
Pb(ii)	−1.2	40		−0.44	93.24			
Zn(ii)	—	2184.6	—	−1.05	—	Glassy carbon electrode	0.1 N HClO_4_	177
Cd(ii)				−0.68				
Pb(ii)				−0.50				
Hg(ii)				+0.45				
Cd(ii)	−0.9	120	100	−0. 71	5.62	Bismuth film microelectrode	ABS & pH = 4. 5	183
Pb(ii)				−0.45	10.36			
Cu(ii)	−1.2	120	4 ×10^3^	—	—	HgO/electrode	0.10 M KNO_3_ and 0.024 M HCl & –	184
Pb(ii)								
Cd(ii)								
Zn(ii)	−1.40	—	—	−1.02	—	HMDE	0.2 M ABS & pH 5.0	185
Cd(ii)		600		−0.62				
Pb(ii)		—		−0.45				
Cu(ii)		—		−0.05				
Cd(ii)	—	120	5–10 × 10^3^	0.022	—	Ultrathin mercury films electrode	0.1 M KNO_3_ + 30 mM HNO_3_ + 0.1 mM Hg^2+^ solution & –	186
Pb(ii)				0.024				
Cu(ii)				0.054				
Zn(ii)	−1.3	600	50	—	1	Metrohm model 6.1246.020 multimode electrode	—	187
Pb(ii)				—				
Cu(ii)				—				
Cd(ii)	−0.85	50	50	−0.65	56.207	Hg/Pt electrode	0.5 M NaCl + HClO_4_ & pH 4.5	188
Pb(ii)				−0.65	103.6			
Cu(ii)				−0.30	—			
Pb(ii)	−1.2	40–280	36.36	—	—	Ta–C : N film electrode	0.1 M KCl & pH 1	189
Cu(ii)								
Hg(ii)								
Pb(ii)	−1.2	240	80–300	—	1.3	AuNPs/SPE	Tris–HCl buffer solution & pH 5.0	190
Cr(iv)	−1.1	180	60	—	0.083	SPE-AuNPs	0.06 M HClO_4_ & –	192
Pb(ii)	−1.3	600	200	−0.4	0.138	Carbon paste electrode (CPE)	0.03 M HClO_4_ & –	193
Pb(ii)	−1.2	240	100	—	82	AuNPs/ionophore/SPE	0.1 M 1Tris–HCl & pH 2	194
Pb(ii)	−0.9	120	20	−0.495	—	Glassy carbon electrode	0.1 M ABS + 0.2 M KNO_3_ & pH 4.50	191
Cu(ii)				−0.019	—			
Zn(ii)	—	600	100	—	1.693	Mercury film electrode (Hg-GCE)	—	196
Cd(ii)					2.91			
Pb(ii)					7.87			
Cu(ii)					1.487			
Zn(ii)	−1.1	60	50	—	1	Metrohm model 6.1246.020 multimode electrode	—	197
Pb(ii)	−1.0	900	5					
Cu(ii)	−1.0	30–300	5					
Hg(ii)	—	120	6	0.54	—	Solid gold electrode	60 mM HCl	198
Hg(ii)				0.62			HClO_4_/NaCl/EDTA	
Pb(ii)	−1.2	137	50	−0.526	—	Carbon disc microelectrode	0.1 M NaClO_4_	199
		198		−0.518				
		259		−0.513				
		319		−0.509				
		379		−0.508				
Pb(ii)	−1.0	900	100	0.1	—	Carbon disc microelectrode	0.1 M NaClO_4_	
Cu(ii)	−0.4	1–150	4	—	—	Gold electrode	0.5 M KNO_3_ & pH 3	200
Pb(ii)	−1.1	900	200	—	0.4144	Boron-doped diamond (BDD) electrode	0.2 M KCl & pH 1.0	201
As(iii)	−3.0	240	30	—	0.19	Gold film glassy-carbon electrode	HCl	202
Cu(ii)	−3.0	300	100	0.05 V	0.006	Chitosan crosslinked with epichloridrin modified CPE	0.1 M KNO_3_ & pH 2.25	203
Cu(ii)	−2	400	—	0	9.3	GCE	Acetate sodium buffer & pH 4.8	204
Zn(ii)				−0.6	45.3			
As(iii)	—	200/250	—	—	2.4	[(BiO)_2_CO_3_-rGO-Nafion]	0.1 M acetate buffer & pH 5.0	205
Cd(ii)					0.8			
Pb(ii)					1.2			

In 1972, the investigation on Cd, Zn, Hg, and Pb ions involved their addition to 0.1 N solutions of HCl, H_2_SO_4_, HClO_4_, and HC_2_H_3_O_2_. HClO_4_ provided the highest possible resolution and sensitivities for the concurrent ascertainment of the targeted MIs. Hence, voltammograms of 0.1 N HClO_4_ containing 5 ppb of Hg, Cd, and Pb ions were acquired regardless of residual current compensation, employing a *t*_d_ of 36.41 minutes (ref. 177). In 2014, Daoli Zhao *et al.* detected heavy metals (Cu^2+^, Pb^2+^, Cd^2+^, and Zn^2+^) both simultaneously and individually by ASV, and in both cases the accumulation time was 120 s, the pH was 4.5, and the working electrode was a CNT thread working electrode.^178^ ASV was used at pH 2 to detect heavy metals in the actual water specimen gotten from Garda Lake, which contained 20 g L^−1^ of Cd^2+^ and Pb^2+^. Employing antimony film modified CPE (SbF-CPE), selecting the value of −1.2 V as the best possible *E*_deposition_.^179^ Applying different preconcentration potentials and deposition times, J. S. Feinberg and W. J. Bowyer used ASV to detect heavy metals. They performed the experiment for each metal in two different scenarios; in one case, they performed the experiment in the presence of a supporting electrolyte, and in another, without an electrolyte. They focused on two metals, namely Pb(ii) and Cd(ii). As a supporting electrolyte, they used 0.1 M KNO_3_, and it was noted that when a supportive electrolyte was present, the stripping peak potential was lower compared to the peak potential obtained without a supporting electrolyte.^180^ Furthermore, ASV was carried out by W. W. Kubiak and J. Wang when other organic surfactants, such as albumin, Triton X-100, Liquinox, and gelatin, are present. They performed the test in three different scenarios for each case: (a) without surfactants and silica, (b) without silica, and (c) with both surfactant and silica. The test solutions they used contained 1 × 10^−7^ M concentration of Zn, Cd, Cu, Pb.^181^ ASV is used to investigate how anodic peak heights for Cu^2+^, Pb^2+^, Cd^2+^, Cu^2+^ Ag^+^, and Hg^2+^ depend on solution pH when poly(ethyleneimine) (PEI) is present Osipova *et al.* applied different *E*_dep_ (depositon potential), *t*_dep_(deposition time), different supporting elecotrolyte with different concentration, and test solution used in this were of different concentration for each metal.^182^ In another study by Baldo *et al.*, employing linear scan mode in ASV, responses were recorded with a bismuth film microelectrode in deaerated ABS (pH 4.5) comprising Cd^2+^ and Pb^2+^ ions with varying concentrations, such as 0.2 × 10^−6^, 0.3 × 10^−6^, 0.4 × 10^−6^, 0.5 × 10^−6^, and 0.8 × 10^−6^ M at deposition voltage = −0.9 V and accumulation time = 120 s. Applying the same conditions, ASV responses were recorded in tap water in linear scan mode at pH = 2. The sample was spiked with Pb(ii) of varying concentrations. *i.e.*, 0.05, 0.1, and 0.3 µM. *E*_d_ and TD were kept at −1.0 V and 300 s, respectively.^183^ With 0.024 M HCl and 0.10 M KNO_3_, accustomed for 300 s at −0.4 V, deposition for 120 s at −1.2 V, and stripped at 2000 mV s^−1^, anodic stripping voltammetric responses were obtained for lake water with the conventional addition procedure.^184^ J. Opydo, in his work for the determination of lead, copper, cadmium, and zinc in soil extracts by ASV, with 2 × 10^−1^ M ABS at a pH value of 5.0 as a base electrolyte, Pb^2+^, Cu^2+^, Cd^2+^, and Zn^2+^ were determined simultaneously from one sample, even though they are in considerably different concentrations. And −0.62, −1.02, −0.05, and −0.45 V, were found to represent the *i*_peak_ of Cd^2+^, Zn^2+^, Cu^2+^, and Pb^2+^ respectively. The concentration of respective metals were, Zn = −0.73 µM; Cd = 0.013 µM; Pb = 0.47 µM; Cu = −2.3 µM (ref. 185). The dynamics of LS anodic stripping voltammetry (ASV) for the concurrent identification of cadmium, lead, and copper was examined at varying rates (50 × 10^−2^ to 10 V s^−1^) and diverse MI concentrations (50 × 10^−3^ to 800 × 10^−3^ µM) employing ultrathin mercury film electrodes by H. P. Wu. As the scan rate rose, all three metals' stripping peaks were seen to migrate toward greater positive potentials, with Cu exhibiting the most alterations over the scan rate range. When comparing Pb and Cd at 10 V s^−1^ to *E*_p_ at 0.5 V s^−1^, for instance, the potential shifts are 22 and 24 mV, as well, whereas for Cu, the same scan rate difference results in a shift of 54 mV (ref. 186). P. J. S. Barbeira *et al.*, in their study on the concurrent measurement of trace quantities of copper, zinc, and lead in rum using LSV, successfully quantified HMIs in the ppb concentration range. The ideal operating parameters for measuring the traces of Zn^2+^, Pb^2+^, and Cu^2+^ samples were deposition voltage −1.3 V, taccumulation = 600 s, anodic scan rate 50 mV s^−1^, and conditioning time 18 s.^187^ A bauxitic soil specimen retrieved from around 4 m deep within the Marghera industrial zone (Venice) was analyzed by S. Daniele *et al.* by acidifying it to pH 4.5, and *E*_peak_ were assigned to Pb (−0.45 V), Cd (−0.65 V), and Cu (−0.30 V).^188^ Lead, copper, and mercury were determined both individually and simultaneously, having different concentrations. Pb(ii) (1 mM), Cu(ii) (20 µM), and Hg(ii) (1.1 µM) were tested in a 0.1 M KCl solution (pH 1), scanning at 36.36 mV s^−1^ individually. While determining simultaneously, a solution containing 8.9 µM Pb ions, 25 µM Cu ions, and 9.2 µM Hg ions was analyzed at pH 1 with a preconcentration time of 40–280 minutes in 0.1 M KCl.^189^ Tukur *et al.* enhanced the ASV experimental conditions by selecting a pH value of 5.0 as the ideal Tris–HCl buffer for determining Pb ions and using a −1.2 V accumulation potential. Higher sensitivity was achieved with a 240 s deposition time. For the Pb(ii) ion analysis, a low LOD (1.3 ppb) was acquired.^190^ Okiei *et al.* measured HMIs in 25 water samples from various coordinates using ASV in a linear sweep; the samples included 160 mg L^−1^ (HgNO3) in 10 × 10^−2^ M ABS with a pH of 4.50. After adding 2 and 0.2 M KNO_3_, everything was well combined. After 10 minutes of N_2_ purging, pre-concentration and stripping were carried out on the solution.^191^ Tukur *et al.* detected Cr(vi) by reducing ions to chromium metal on SPE-AuNPs using a −1.1 V accumulation voltage for 180 seconds. They determined the metal ion with a LOD of 1.6 × 10^−3^ µg L^−1^.^192^ Mouhamed *et al.* also optimized the experimental parameter by applying different conditions for each parameter in the measurement of Lead in Water using Unmodified CPE. The method yielded a LOD of 13.8 µg L^−1^ for Pb ions.^193^ Under optimized set-up [deposition voltage −1.2 V, preconcentration time 240 s, scanning at 0.1 V s^−1^, and Tris–HCl (at pH 2, 10 × 10^−2^ mol L^−1^)], the anodic stripping voltage–current graph at −0.05 × 10 V was correlated to the concentration. Of Pb^2+^ within the bounds of 0.4 to 20 mg L^−1^ Pb(ii) with an LOD of 0.82 mg L^−1^.^194^ ASV has also been employed in 2024 in the investigation of Cu(ii) and Zn(ii) in Pig Farm Wastewater. The investigation employed a glassy carbon electrode under optimum conditions, which was found to be – the deposition potential of −2 V, enrichment time of 400 s, pulse period 0.03 s, pulse width of 0.6 s, pH 4.8, and acetic acid-sodium acetate buffer. Under such conditions, LOD of 9.3 µg L^−1^ and 45.3 µg L^−1^ were obtained for Cu(ii) and Zn(ii), respectively.^195^

### Cyclic voltammetry

Cyclic voltametry has been utilized for the purpose of detecting HMIs in a quick and selective manner.^206^ Due to exposure to industrial processes and modernization, heavy metals pose a serious health risk. Heavy metal poisoning of food, water, and air is a worldwide issue that affects millions of people. There are gender differences in metal toxicity. While low-dose exposure may cause neuropsychiatric issues, high-dose exposure might have major consequences.^207^ Thus, for the detection of HMIs, cyclic voltammetry is a significant electrochemical method, giving excellent sensitivity, selectivity, and the capability for concurrent detection of several metals.^208^ It is a prevalent electrochemical technique for examining molecular entities' reduction and oxidation reactions.^209^ An extremely effective general method for the electrochemical analysis of coatings on conductive surfaces and trace quantities of compounds in water.^210^

Cyclic voltammetry (Table 4) involves sweeping the voltage applied to an electrochemical cell back and forth between two set potentials while measuring the resulting electric current due to oxidation–reduction reactions happening at the exterior of the WE.^211^ The voltage of the WE is linearly ramped up and down cyclically while recording the current at the electrode.^210^ These potential cycles are repeated until a cyclic steady state is reached by the voltammetric trace.^212^ Cyclic voltammetry uses a 3-electrode setup comprising a reference, counter, and a working electrode, just like other voltammetric methods.^213^ A triangle voltage or other periodic potential is applied between the WE and CE. Then, at the WE, the analyte undergoes oxidation and reduction.^214^

**Table 4:** Optimized experimental parameters and analytical performance for detecting heavy metal ions by cyclic voltammetry

Heavy metals	Voltage range (V)	Scan rate (mV s^−1^)	Peak potential (V)	Detection limit (µg L^−1^)	Supporting electrolyte	Working electrode	Buffer and pH	Ref.
Hg(ii)	−0.2 to1	100	—	0.2	—	Graphene modified GCE	25 mM tris–HCl buffer & –	217
Pb(ii)	−1 to 0	50	−0.67 (*E*_peak_^c^)	0.186	—	Covalent binderless bulk modified electrode. (Bulk modification of graphitic carbon with 4-aminosalicylic acid)	Sodium, acetate, solution & pH 8	219
Cd(ii)			−0.87 (*E*_peak_^c^)	1.203	—			
Cd(ii)	0.2 to 0.6	50	—	0.049	—	Immunodyne membrane-HRP	0.1 M ABS & pH 5	220
Cu(ii)	−0.3 to 0.8	50	—	16.522	0.2 mM HCl	PU/Pt modified electrode	—	221
Hg(ii)	−0.6 to 1.2	—	—	0.2347	—	PUU-Au/CPE	0.03 M phthalic acid & pH 2.5	222
Cd(ii)	—	100	−0.67 (*E*_peak_^c^)	8992.8	0.1 M KCl	AC/GCE	—	223
Hg(ii)	−0.1 mV to 0.6 mV	50	0.10 × 10^−9^ (*E*_peak_^c^)	40.12–60.18	0.5 M H_2_SO_4_	Montmorillonite-modified Pt electrode	—	224
			0.05 × 10^−3^ (*E*_peak_^a^)			Kaolinite-modified Pt electrode		
Cu(ii)			−0.09 × 10^−3^ (*E*_peak_^a^)			Montmorillonite-modified Pt electrode		
			0.01 × 10^−3^ (*E*_peak_^a^)			Kaolinite-modified Pt electrode		
Cu(ii)	0.75 to −1.5	200	0.1 (*E*_peak_^a^)	—	—	Pt wire	– & pH 7	225
Pb(ii)	–-1.5 to +1.5	100	—	1.158	0.1 M NaCl	Carbon-paste electrode CPE-1% EDTA	– & pH 2	226
Hg(ii)				0.828				
Cd(ii)				0.571				
Co(ii)				0.07				
Pb(ii)	−1.0 to 0.2	0.1 to 0.5 V s^−1^	—	—	0.1 M KCl	Blast furnace slag modified GCE	—	228
Hg(ii)	−0.5 to 0.5	50	—	—	—	1,10-Phenanthroline drop cast on the carbon electrode	—	229
Pb(ii)	−1 to 0		—			QDPPZ drop casted on carbon electrode		
Pb(ii)	0.00 to 0.50	10–250	0.1	—	—	Au/GO	—	230
Cd(ii)	−2 to 1	0.01 to 1 V s^−1^	−0.7	10.91	—	AC-reduced graphene oxide (RGO/CPE)	– & pH 3	233
Pb(ii)			−0.4	14.01				

Typically, glassy carbon, gold, or platinum is used to make the working electrode. Pt electrodes are usually employed as counter electrodes, although reference electrodes, such as electrodes based on the redox couple Ag/Ag^+^, may be used. There are basically two options for the sample: either the analyte dissolves in the electrolyte, or it is solidly accumulated on the WE. Where the processes are reversible, and the analyte is dissolved in the electrolyte, the *E*_1/2_ should match the potential of the redox pair, which is expressed by the reduced and oxidized species.^209,214^ The current outcome is associated with the rate of the redox process, which is the forward or reverse transfer of electrons at the working electrode.^215^

It is possible to use and then modify the Randles–Evčík equation to measure the analyte concentration, which is
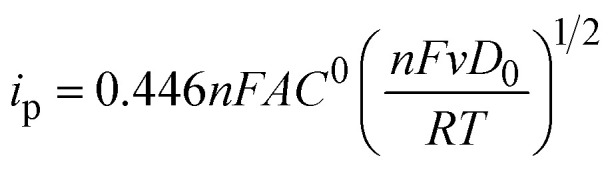
Here in the redox event, *n* refers to the no. of electrons exchanged, *D*_0_ (cm^2^s^−1^) represents the diffusion coefficient, *C*^0^ expresses the analyte's bulk concentration, and *A* (cm^2^) represents the electrode surface area. The *i*_peak_, *A*, rises linearly with the square root of the scan rate *υ* (Vs^−1^).^209,216^

The successful quantification of Hg^2+^ through the utilization of CV has been accomplished by Talat *et al.*, with a LOD of 0.201 µg L^−1^.^217,218^ Kempegowda *et al.* employed this technique to detect Pb^2+^ and Cd^2+^ at trace level applying pH 8 and scan rate 50 mVs^−1^ (ref. 218 and 219). The cyclic voltammetric method was employed for detecting cadmium ions in drinking water, resulting in a detection limit of 50. To control pH, a 0.01 M ABS solution was used.^220^ A study was found where Cu(ii) was detected with a 16.72 ng mL^−1^ detection limit conducted by El-Raheem *et al.*,^221^ and in another, El-Raheem *et al.* spotted Hg^2+^ with a LOD of 0.235 µg L^−1^,^222^ both studies were done under the CV technique. At a scan rate of 100 mVs^−1^, a Pt wire, 0.1 M potassium chloride as a supporting electrolyte, and silver/silver chloride (3 M NaCl) as a counter and reference electrode were utilized to sense Cd(ii). And a span of oxidation current (5.5598 to 2779.9) µA was obtained for a 0.008–0.1 mM concentration range of Hg(ii) analyte.^223^ A. Issa *et al.* used CV at conditions, where the scan rate was 50 mV s^−1^ and 0.5 M H_2_SO_4_ solution, peak oxidation current, and their corresponding concentration of Hg^2+^ and Cu^2+^ for different working electrodes were obtained. For Hg(ii), at a concentration of 2.0 × 10^−6^ M, 11.3 uA and 16.0 µA peak oxidation current were observed for the montmorillonite-modified Pt electrode and the kaolinite-modified Pt electrode, respectively. And for Cu(ii), at a concentration of 1.0 × 10^−4^ M, 50 µA and 20 µA peak oxidation current were observed for the montmorillonite-modified Pt electrode and the kaolinite-modified Pt electrode, respectively.^224^ Ramadhan *et al.* employed cyclic voltammetry for the detection of Cu(ii), utilizing various circumstances, including pH fluctuations from 4 to 8 and voltage adjustments ranging from 0.75 V to −0.75 V, −1.0 V, −1.25 V, and 1.5 V. The reaction during the process

7 Co(s) ⇌ Co^2+^ + 2e^−^

8 Co^2+^ + 2e^−^ ⇌ Co(s)

**Fig. 5:**
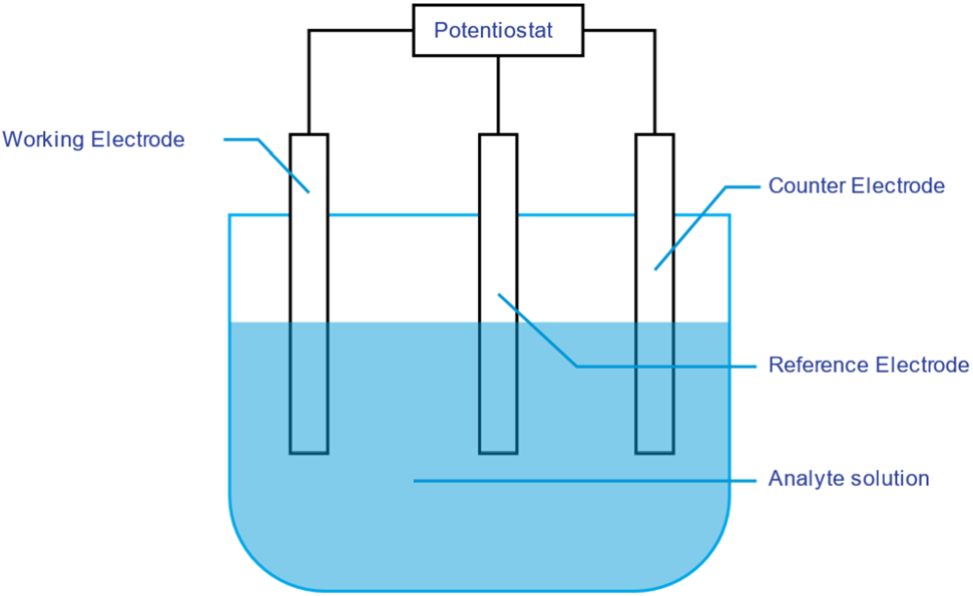
Instrumentation in voltammetry.

It was noted that, scanning at 200 × 10^−3^ V s^−1^, detection was highly feasible. A chemical waste solution with an indeterminate concentration of a mixed residual solution was analyzed. A notable signal at 0.1 V indicates the presence of cobalt, with a concentration of 648.4 ppm in the sample.^225^ Pb^2+^, Cd^2+^, Hg^2+^, and Co^2+^ were analyzed at a concentration of 0.3 × 10^−3^ mol L^−1^ using EDTA modified CPE with a LOD of 1.16, 0.46, 1.02 and 0.07 µg L^−1^, respectively in tap water (in Morocco).^226^ Additionally, S. Deshmukh *et al.* used this technique to find HMIs in water.^227^ At the same time, detecting Pb(ii), a 0.1 M KCl solution was utilized as a supporting electrolyte. Regression line equation derived from the plot of the square root of scan rates *vs.* current is *y* = 2.227*x* + 0.763 and *R*^2^ = 0.990, with the conc. of Pb^2+^ being 5 mM (ref. 228). A reduced *E*_peak_ of 0.2 eV was observed for Hg(ii) while scanning at 50 mV s^−1^, with the 1,10-phenanthroline sensor. And a Reduction of −0.6 eV for Pb(ii) was recorded at a scan range of -1 V to 0 V and 50 mV s^−1^ scan rate with QDPPZ sensor.^229^ O. Surucu employed CV ranging from 0.00 V to +50 × 10^−2^ V in three distinct water samples (well, rural, and urban water) in his trace analysis of heavy metals. As it could be seen, one distinct oxidation peak at +10 × 10^−2^ V and one distinct cathodic peak at +20 × 10^−2^ V indicated the heavy metal ions content of well water.^230^ Using CV, F. Vajedi and H. Dehghani determined the concentrations of Cd^2+^, Pb^2+^, and Cu^2+^ by characterizing TiO_2_ nanostructures and TiO_2_/rGO nanocomposite (TG3) electrodes. They utilized an aqueous solution of 0.10 M PBS (pH 7.4) to regulate the pH, and the settings they utilized were: a voltage range of −1 to 2 V, scanned at 10 × 10^−3^ V s^−1^ for 30 seconds. An anodic peak at approximately −1.56 V and a reduction peak at around −0.48 V can be seen for the TiO_2_ electrode. Potential shifts to 1.4 V and −0.37 V are shown by the TG3/GCE anodic and cathodic peaks, respectively.^231^ A recent (2024) investigation on divalent cadmium and lead ions in wastewater was conducted through the use of CV. The investigation utilized a modified carbon paste electrode (mCPE). Three modified versions (AC-Graphite modified mCPE, AC-Reduced Graphene Oxide (RGO) modified mCPE, and AC-RGO-Chitosan modified mCPE) were utilized. Among them, AC-RGO/mCPE demonstrated the highest recovery percentage (99.641 ± 1.286) for Cd(ii) and (99.132 ± 0.464) for Pb(ii). Also demonstrated the lowest RSD values, *i.e.*, 3.96% for Cd(ii) and 7.01% for Pd(ii). The measurements were carried out at a pH of 3, potential window −2 V to 1 V, and scan rate 0.1 V s^−1^ throughout the study (Fig. 5).^232^

### Differential pulse voltammetry (DPV)

DPV is an effective electrochemical practice for detecting HMIs owing to its inherent sensitivity, low LOD, and the ability to concurrently detect many HMIs. The affordability, heightened sensing ability, and remarkable mobility of this approach render it an effective method.^234,235^ This technique was employed to evaluate the electrical signals of HMIs.^236^ In short, it's a method that leverages a succession of pulses to the electrode with a steadily increasing baseline voltage. It helps to minimize the effect of background charging current during analysis.^237^ Every pulse has its current measured twice: right before it is applied and right after it is finished. The disparity between the two recorded currents for every pulse is graphed against the baseline potential.^238,239^ Therefore, the name “differential” voltammetry. Some frequent applications of this are immunoassays and HMI identification and quantification.^239^ Voltammetric techniques such as DPV (Table 5) are suitable for trace and ultra-trace HMIs; they require comparatively reasonably priced instrumentation and have exhibited proficiency in multi-metal study.^240^

**Table 5:** Optimized experimental parameters and analytical performance for detecting HMIs by DPV

Heavy metal	Scan range (V)	Pulse amplitude (mV)	Scan rate (mV s^−1^)	Peak potential (V)	Limit of detection (LOD) (µg L^−1^)	Working electrode	Electrolytes & pH	Ref.
Pb	−0.9 to 0.1	25.05	—	0.45	—	Glassy carbon modified	HCl medium	268
Cd				0.65				
Cu(ii)	—	100	10	—	57.128	CT-GCE	Phosphate buffer solution (PBS) & pH 7	243
Pb(ii)					64.025			
Cd(ii)					264.164			
As(iv)					49.973			
Pt(IV)					6.828			
Cr(iii)	—	—	—	−1.25	9879.259	Hg-SPCEs	Acetic-acetate buffer & pH 4 solution	246
Cr(vi)				−0.1	249.581	AuNPs-SPCEs		
Cd(ii)					0.095	Glassy carbon electrode	0.1 M PBS & –	249
Cu(ii)	−0.8 to +0.1	50	50	−0.5	14	SPCE/AuNP/CD-modified electrode	Phosphate buffer solution & pH 2.0	250
Pb(ii)				0.0	4.2			
Cd(ii)				−1.0	2.8			
Pb(ii)	—			−1.0	48	[Ru(bpy)_3_]^2+^/graphene/Nafion		251
Cd(ii)		0.0025			49			
Cu(ii)					28			
Hg(ii)		50		0.05	0.03	Screen-printed carbon electrode (SPCE)	(HCl 0.1 M) & pH 7.0	252
Pb(ii)	—	—	—	−0.5	10.36	R-CMEs	0.1 M acetate buffer solution & pH = 4.5	253
Cd(ii)	−1.0 to −0.2	50	—	−0.8	0.022	CNTs-UiO-66-NH_2_ modified GCE	Acetate buffer solution & (pH 5.0)	254
Sn(ii)	−0.25 to −0.55	−100	4	−0.446	—	—	0.04 M acetic/*o*-phosphoric/boric acids	255
Pb(ii)				−0.346	—	—		
Hg(ii)	+ 0.3 to − 0.6	50	0.05	—	—	Gold electrode	Acetate buffer solution & (pH = 4.5)	256
Cd(ii)	−1.0 to 0.4	50	—	−0.88	—	PA/PPy/GO modified electrode	0.1 M acetate buffer solution & (pH 4.5)	257
Pb(ii)				−0.64				
Pb(ii)	—	20	—	−0.185	0.315	NH_2_-MIL-53(Al)/PPy modified electrode	0.1 M acetate buffer solution & (pH = 5.0)	258
Cu(ii)				0.20	0.244			
Cu(ii)	—	90	—	—	0.02	Modified glassy carbon electrode	—	259
Pb(ii)					0.03			
Cr(iii)					0.5			
Ag(i)	—	50	—	+0.814	3.236	CNTs/GC electrode	0.1 M acetate buffer solutions & (pH 5.0)	260
Cd(ii)	—	—	—	−0.834	2.023	Poly(BPE)/g-C_3_N_4_	0.1 M acetate buffer solution & pH = 4.5	261
Pb(ii)				−0.586	0.671			
Cd(ii)				—	1.09			
Pb(ii)				—	0.677			
Pb(ii)	—	—	100	—	6.2	Modified COOH–C4	0.1 M KCl & (pH 7)	262
Hg(ii)	−0.2 to +0.5	100	50	—	0.2	Magnetic, carbon, paste, electrode (MCPE)	0.1 M HCl & pH 3.5	263
Cd(ii)	—	50	—	−0.8	0.1	Bi/LC-rGO/DSPE	0.1 M acetate buffer solution & (pH 4.5)	264
Pb(ii)				−0.6	0.08			
Cd(ii)	−1 to 1	90	15	−0.85	111.29	Gold nanoparticle-modified carbon thread electrodes	HCl–KCl buffer & pH 2	269
Pb(ii)				−0.60	128.46			
Cu(ii)				−0.20	87.70			
Hg(ii)				0.20	144.42			
Cd(ii)	—	—	50	−0.75	1.13	Arc-ferrite/N-rGO/CPE	Acetate-KCl buffer & pH 4.5	270
Pb(ii)				−0.51	1.01			
Hg(ii)				0.12	1.07			
Bi(iii)	−0.8 to 0.6	30	—	—	0.65	CoWO^4–^CoMn_2_O_4_/nitrogen-doped graphene/CPE	0.1 M HNO_3_–NaNO_3_ buffer & pH 1.0	271
Pb(ii)					0.52			
Hg(ii)					0.12			
Cd(ii)	—	—	100	—	0.12	NaAlO_2_/CPE	Britton–robinson buffer & pH 5	272
Cd(ii)	−1.5	300	—	−0.86	9.4	AuNPs-SPCE	HCl treated tap water	273
Pb(ii)				−0.56	4.4			
As(ii)				−0.02	7.6			
Hg(ii)				0.1	1.5			
Cd(ii)	−0.8	50	25	−0.19	13.84	MnCo_2_O_4_/PAA-jute fibers	H_2_SO_4_/KCl buffer & pH 2	274
Pb(ii)				−0.36	0.756			
As(iii)	—	100	200	—	3.0	Screen-printed graphene electrode (SPGE)	0.1 M HCl	275
Cr(vi)				—	40			
Hg(ii)				—	16			
Cd(ii)				−0.9	2.0			
Pb(ii)				−0.68	0.95			
Cd(ii)	—	0.05	50	0.95 V	0.095	LSG/PB-PEDOT/GCE	0.1 M acetate buffer at pH 5	276

Jin *et al.* under optimum experimental conditions used PdNPs/g-C3N4/GC as a working electrode to determine Hg^2+^ ion. An LOD of 0.009 µg L^−1^ was recorded for Hg^2+^. The output was noted at a scan span of −0.2 V to 0.6 V, with the height of pulse, step potential, and frequency set as 0.250 × 100 mV, 0.004 × 10^3^ mV, and 5 Hz, respectively.^241^ This technique was employed to quantify the concentration of mercury ions.^242^ Zn and Mn were analyzed in 0.01 M NH_3_ + 0.01 M NH_4_Cl base solution by Jin *et al.* with the DPV method. Peak potentials of Zn and Mn were recorded at −1.175 V and −1.495 V, respectively.^240^ Huitle *et al.* obtained detection ranges for Cu^2+^ from 3.99 to 39.1 µM, Pb^2+^ from 1.99 to 15.8 µM, Cd^2+^ from 15.9 to 62.3 µM, and Co^2+^ from 611 to 2780 µM. The detection limits for each metal were 0.3(Pb), 0.899 (Cu), 2.35 (Cd), and 0.667 (As) µM.^243^ The concentration of Pb ions was measured between 5 × 10^−4^ M and 8 × 10^−7^ M, yielding a *R*^2^ value of 0.970 (ref. 244). A diamond electrode was put into action in a study to detect MIs over a voltage span of +0.08 × 10 V to −0.004 × 100 V. The experiment was run with a pulse width of 50 × 10–3 s, a pulse height of 25 × 10–3 V, and a scan rate of 5 × 10^−3^ V s^−1^.^245^ Cr(iii) was determined by using Hg-SPCEs electrode, and accumulation was executed at a voltage of 0.9 V for 3 minutes. The peak potential was observed at −1.25 V. Cr(vi) was determined by using an AuNP-SPCE with a peak potential of −0.1 V.^246^ Under ideal conditions, the LOD for Cd ions was recorded at 1.10 nM, exhibiting an extensive dynamic linear range of (0.2 × 10^−7^ to 20 × 10^−6^) M and (20 × 10^−6^ to 900 × 10^−6^) M.^247^ Liu *et al.*, in their work, used an ion liquid/reduced graphene oxide composite GCE for concurrent analysis of Pb and Cd ions. The LODs were 0.71 ng mL^−1^ and 0.58 ng mL^−1^, respectively.^248^ Under ideal circumstances, cadmium was directly and readily identified in drinking water and sewage samples, with a low LOD of 0.85 nM (ref. 249). Pudza *et al.* applied DPV for the sensitive and selective determination of Cu, Pb, and Cd. The LOD recorded for Pb^2+^, Cd^2+^, and Cu^2+^ were 0.0042, 0.0028, and 0.014 ppm, respectively. DPV was set at a voltage range of −0.8 to +0.1 V, pulse width of 50 × 10^−3^ s, scanning at 50 × 10^−3^ V s^−1^, and pulse rate of 0.2 V.^250^ Using [Ru (bpy)_3_]^2+^ and a graphene-modified electrode, Pb^2+^, Cd^2+^, and Cu^2+^ were detected with the detection limit of 48, 49, and 28 ng mL^−1^, respectively.^251^ In another experiment, the parameters were set at 15 × 10^3^ µV for the step potential and a pulse height of 50 × 10^−3^ V, with 5 ppm Hg on SPCE. An anodic peak at 50 × 10^3^ µV was detected. The detection limits and linear range were determined to be 30 × 10^−3^ ng L^−1^ and 0.1–30 ppb, respectively.^252^ Employing DPV, cadmium, lead, copper, and mercury were analyzed with poly R-modified electrodes. In the anodic scans, peaks for respective HMIs dissolution were noticed at the voltage of −0.762 V, −0.532 V, −0.078 V, and +0.301 V, respectively.^253^ Yu *et al.* used modified GCE electrodes in ABS comprising 100 µM Cd ions at a fixed voltage of −0.013 × 100 V for 2.5 minutes. The linear detection range for cadmium(ii) was determined to be between 0.3 × 10^−3^ M and 150 × 10^−3^ M, with a LOD of 20 × 10^−8^ M (ref. 254). Sabry *et al.* conducted a concurrent analysis of Sb and Pb in soft drinks, recording the voltammograms of both ions within a potential spanning between −0.25 to −0.55 V, utilizing a pulse height of −0.1 V, and a 4 × 10^−3^ V s^−1^ scan rate.^255^ For the detection of Hg, the experimental conditions were set at: height and width of pulse are 0.05 V and 50 ms, scanning rate 0.0005 × 100 V s^−1^, the preliminary voltage +0.3 V, and the concluding voltage −0.6 V.^256^ Dai *et al.* employed a PA/PPy/GO modified electrode to quantify Cadmium and Lead within a linear range of 5–150 ppb, utilizing a pulse breadth of 0.05 s, a deposition potential of −1.2 V, a pulse period of 0.2 s, a pH value of 4.5, and an amplitude of 0.05 V. The maximum potentials of Cd^2+^ and Pb^2+^ were noted at 0.88 V and 0.64 V, respectively.^257^ Wang *et al.* utilized an NH_2_-MIL-53(Al)/PPy modified electrode, demonstrating effective sensing capabilities for the quantification of Copper and Lead within the scale of 1–400 ng mL^−1^. The observed detection limits were 244 × 10^−3^ µg L^−1^ and 315 × 10^−3^ µg L^−1^. The measurements were completed at a pulse width of 5 × 10^−2^ s, height of 20 × 10^−3^ V, period of 0.2, and a quiet duration of 2 s in a 10 × 10^−2^ mol L^−1^ ABS at pH 5.0 (ref. 258). For the measurement of chromium, lead, and copper in real food and water, Ghorbani *et al.* used this method. Detection limits of 0.02, 0.03, 0.5 ng mL^−1^ and linear range of 0.08–570, 0.1–1200, 0.5–1300 ng mL^−1^ were observed, respectively.^259^ This technique was used between 0.6 V and 1.1 V, with a 0.004 V increment potential, a 0.05 s pulse breadth, a 0.05 V pulse height, and a 0.2 s pulse length. The detection limit for Ag(i), relying on the Ag^+^-G sensory mechanism, was 30 × 10^−9^ M, with a linear range of 10 × 10^−8^ M to 25 × 10^−7^ M (ref. 260). Ding *et al.* in their work, used a poly(BPE)/g-C_3_N_4_ composite-modified electrode for the concurrent determination of Cd and Pb, with detection limits of 0.018 × 10^−6^ M and 0.00324 × 10^−6^ M, respectively. The experiment was performed by deposition at −1.4 V, and applying a potential interval of −1.4 to −0.2 V, a pulse width of 50 milliseconds, and an accumulation duration of 3 minutes and 30 seconds. Whereas, for individual determination of Cadmium and Lead, the LOD of 1.09 µg L^−1^ and 0.00068 µg L^−1^ were found respectively under optimal conditions.^261^ Aziz *et al.* applied the DPV technique with a modified COOH–C_4_ electrode for detecting Pb^2+^, and the LOD of 6.2 ppm was noted.^262^ Fayazi *et al.*, in their work, detected Hg(ii) using DPV with a LOD of 0.2 µg L^−1^. The DPV responses were recorded using a pulse time of 0.04 s, a height of 0.1 V, and a step increment of 4 millivolt between −0.2 and +0.5 V.^263^ Under optimized parameters, Bi/LC-rGO/DSPE was used to analyze trace amounts of Pb and Cd in decorative material. The detection limits observed for respective ions were 0.1 µg L^−1^ and 0.08 µg L^−1^, respectively.^264^ MnCo_2_O_4_/acrylic acid grafted Jute fibers were utilized as an electrochemical sensor to detect Pb(ii) and Cd(ii) in aqueous solution through differential pulse voltammetry. Under the optimized condition, two anodic peaks −0.36 V for Pb(ii) and −0.19 V for Cd(ii) were observed, and detection limits were calculated to be 13.84 µg L^−1^ and 0.756 µg L^−1^ for Cd(ii) and Pb(ii), respectively.^265^ DPV was also used in 2025 for the simultaneous detection of Cd(ii), Pd(ii), Cu(ii), and Hg(ii) in water samples. Real water samples from various lakes in Hyderabad, India, were analyzed, which demonstrated a linear range of 1–100 µM. With gold nanoparticles deposited on carbon threads, the accuracy of the model was found to be 99%. The resultant LOD for Cd(ii), Pd(ii), Cu(ii), and Hg(ii) were calculated to be 0.99, 0.62, 1.38, and 0.72 µM (ref. 266). In 2026, DPV was employed for high-sensitivity detection of HM in water using Arc-ferrite/N-rGO nanocomposites as the electrochemical sensor. The optimal conditions were established as follows: an acetate-KCl buffer (pH 4.5), a deposition potential of −1.1 V, a scan rate of 50 mV s^−1^, and a 180 s accumulation time. The model demonstrated LOD of 1.13, 1.01, 1.07 ppb for Cd(ii), Pd(ii), and Hg(ii), respectively, also a linear range of 10–150 ppb.^267^

### Comparative overview of voltammetric techniques: advantages and limitations

DPASV offers several benefits, including as low cost, excellent sensitivity, and short analysis time, and the capability for simultaneous element detection compared to other methods.^277^ It can detect extremely low concentrations, even in high salt content environments, and supports the determination of metal speciation by distinguishing between free and complexed metal ions. This technique can also be used to estimate non-metals like organics or anions.^278^ Additionally, it is thought to be quick, easy, selective, and affordable for heavy metal analysis, both qualitatively and quantitatively.^279^ However, limitations include the accumulation of organic matter or interfering substances on the electrode surface, which can affect measurement reproducibility and accuracy. Sample matrices with varying pH, ionic strength, or surfactants can influence stripping signals, requiring optimization.^280,281^ Additionally, DPASV requires a preconcentration phase, increasing total measurement time.^282^ SWASV provides quick and accurate detection of metal ions with low LOD as 10^−9^ M, (ref. 283) and it does not require oxygen removal, simplifying procedures.^284^ Its limitations involve the need for precise parameter optimization, such as frequency, amplitude, and step potential, to ensure accuracy,^283^ Its response can be influenced by variations in electrode surface morphology.^285^ Anodic Stripping Voltammetry (Linear ASV) is simple and cost-effective, especially in low-resource settings, and allows simultaneous measurement of metals like Cd, Pb, Cu, and Zn.^280,286–288^ Nonetheless, it has lower sensitivity than pulse-based methods, resulting in higher noise and reduced peak resolution,^289^ and requires longer deposition times, which is a drawback for high-throughput analysis.^290^ Cyclic voltammetry (CV) is advantageous for studying redox processes, reaction mechanisms, and electrode kinetics, as well as for developing sensor materials.^291,292^ However, it has low sensitivity for trace analysis, lacks selectivity in multicomponent samples due to overlapping peaks, and is not optimized for quantitative measurements.^293–295^ Lastly, differential pulse voltammetry (DPV) offers high resolution and sensitivity, is suitable for multi-analyte detection, and produces lower background currents, improving signal quality for low-concentration samples.^296,297^ Its drawbacks include the need for regular electrode maintenance, slower operation compared to SWV, and the necessity for optimized parameters such as pH, electrolyte, and scan rate.^296,296,298^

### Critical discussion, comparative literature analysis, and practical implications

The research under evaluation shows that voltammetric methods are very successful in detecting heavy metal ions (HMIs) in wastewater in a sensitive and selective manner. Due to the addition of a preconcentration step, which greatly improves sensitivity and selectivity, square wave anodic stripping voltammetry (SWASV) and differential pulse anodic stripping voltammetry (DPASV) demonstrated superior analytical performance among the methods investigated. Ultra-low detection limits for Pb(ii), Cd(ii), Hg(ii), and Cu(ii) have been reported in a number of studies, especially when using nanostructured electrodes like graphene, carbon nanotube, and metal–organic framework-based electrodes.^102,131,141,167^

A deeper examination of the results shows that optimal operating parameters, such as deposition potential, deposition time, pH, pulse amplitude, and supporting electrolyte composition, have a significant impact on analytical performance. For effective metal buildup and stripping, the majority of investigations found that slightly acidic conditions (pH 4–5) and deposition potentials between −1.0 and −1.4 V were ideal,^63,74,134^ These findings imply that the electrolyte environment and electrode surface properties are important factors in electron-transfer kinetics and sensor sensitivity.

The results are in line with other research showing that voltammetric approaches offer notable benefits over traditional spectroscopic techniques like AAS, ICP-MS, and ICP-OES. Spectroscopic methods are quite accurate, but they need expensive equipment, complicated sample preparation, and experienced operators,^37,38,39^. Voltammetric techniques, on the other hand, are quick, affordable, portable, and appropriate for on-site environmental monitoring.^41,42^ The current review also emphasizes how advanced electrode modifications significantly enhance selectivity, repeatability, and detection capabilities, supporting previous electrochemical sensing investigations.^139,160^ The progressive replacement of mercury electrodes with eco-friendly substitutes, such as carbon-based electrodes, bismuth-film, and antimony-film, is another significant finding. These green electrode materials can achieve similar or even better analytical performance while lowering environmental toxicity concerns, according to recent studies.^123,142,154^

**Fig. 6:**
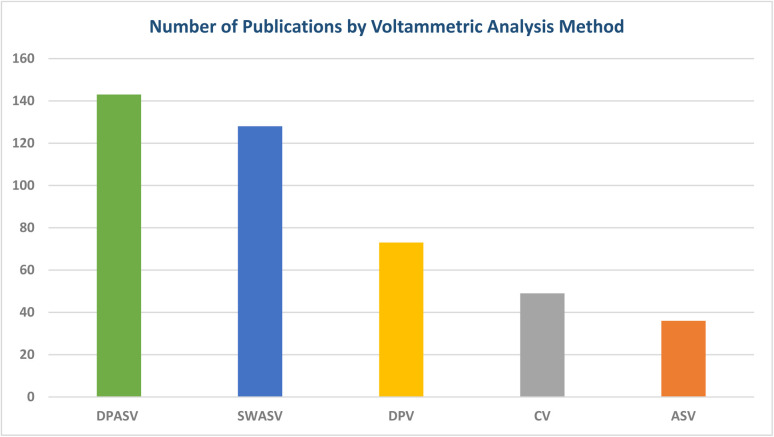
Number of publications by the voltammetric analysis method till 2026.

**Fig. 7:**
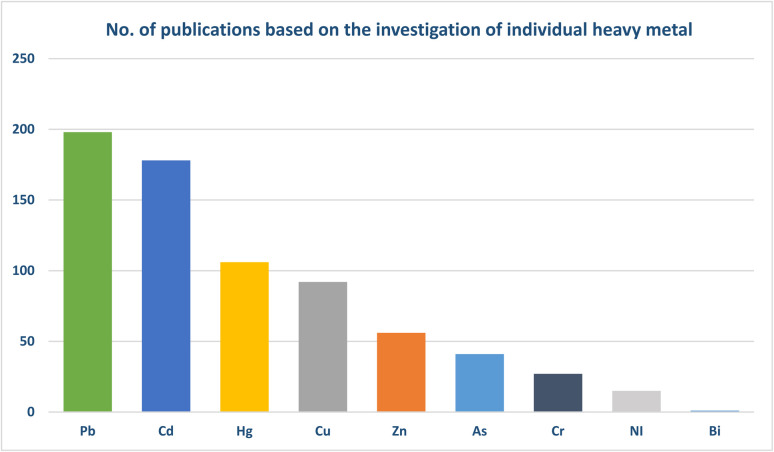
Number of publications based on investigation of individual heavy metal by voltammetric techniques till 2026.

These results have important practical ramifications for wastewater treatment plants, environmental monitoring groups, and businesses. SWASV and DPASV's low detection limits and quick analysis capabilities allow for the early identification of hazardous metals in industrial effluents, assisting businesses in lowering ecological risks and enhancing environmental compliance. Additionally, portable voltammetric sensors provide a cost-effective way to monitor in real time, especially in areas with limited resources. In general, voltammetric methods offer sensitive, practical, and sustainable ways to regularly monitor heavy metals in wastewater systems (Fig. 6 and 7).

## Conclusion

In the field of electrochemical analysis, Voltammetry have become an important tool. Especially for heavy metal detection, as they offer a low detection limit, a higher percentage of recovery, a lower time requirement, cost-effectiveness, and portability. This review covers SWASV, DPASV, ASV, CV, and DPV for the quick and accurate detection of multiple HMIs selectively and sensitively. Among them, DPASV and SWASV stand out because they show enhanced sensitivity. SWASV, DPASV, and ASV, they all incorporate a preconcentration step to improve detection accuracy. However, SWASV and DPASV show distinct advantages. SWASV minimizes capacitive current, giving better peak resolution, while DPASV reduces background noise and enhances analytical precision. The review of “different types of voltametric techniques for selective and sensitive detection of HMIs in wastewater system” has significant promise for the development of heavy metal electrochemical detection. By comparing different voltametric techniques systematically, it certainly provides critical insights for the researchers to select the optimal methods with greater sensitivity and selectivity. The findings this review offers has strong environmental and industrial relevance as it will aid in wastewater treatment and regulatory compliance. In addition, discussion of sensitivity, selectivity and detection limits contributes in advancing analytical chemistry. By bridging research with practical applications, it serves as a important resource for both academia and industry.

## Author contributions

Md. Shadat Hossain and Md. Habibur Rahman collected the data and wrote the draft and original manuscript. Md. Sahadat Hossain conceived and designed the review, supervised and analyzed the data. Md. Kawcher Alam and Md. Tariqur Rahaman Bhuiyan assisted in collecting data. Md. Kawcher Alam supervised and assisted in collecting data.

## Conflicts of interest

There are no conflicts to declare.

## Data Availability

No data were generated for this article.
